# Lutein and Zeaxanthin in the Lipid Bilayer–Similarities and Differences Revealed by Computational Studies

**DOI:** 10.3389/fmolb.2021.768449

**Published:** 2021-10-26

**Authors:** Krzysztof Makuch, Jakub Hryc, Michal Markiewicz, Marta Pasenkiewicz-Gierula

**Affiliations:** Department of Computational Biophysics and Bioinformatics, Faculty of Biochemistry, Biophysics and Biotechnology, Jagiellonian University, Krakow, Poland

**Keywords:** xanthophyll, polar interactions, conformational freedom, molecular dynamics, transmembrane rotation, interaction lifetimes

## Abstract

Lutein and zeaxanthin are two similar carotenoids of the xanthophyll subgroup. Carotenoids are synthesized almost entirely by plants but are also present in significant amounts in animals. They are essential components of the lipid matrix of biomembranes, and one of their functions is to protect cells from light radiation, free radicals and oxidative stress. Carotenoids, depending on their chemical structure, can locate at various positions and in different orientations in the bilayer. Xanthophylls (XAN) are polar and in the bilayer are positionally restricted. In the case of lutein and zeaxanthin, whose both ionone rings are hydroxy-substituted and as such are anchored in the lipid bilayer interfaces, the position is generally transmembrane. However, both experimental and computer modelling studies indicate that lutein can also locate horizontally below the bilayer interface. This location has never been observed for zeaxanthin. To find a molecular-level explanation for the difference in the orientations of the XAN molecules in the bilayer, a number of phosphatidylcholine-XAN bilayers were constructed and molecular dynamics (MD) simulated for 1.1 µs each. The all-*trans* XAN molecules were initially placed either parallel or perpendicular to the bilayer surface. With the exception of one lutein, the horizontally placed molecules adopted the transmembrane orientation within 100–600 ns. On the basis of detailed analyses of the XAN orientations and the numbers and lifetimes of their interactions in the bilayer, a plausible explanation is offered as to why a lutein molecule may remain in the horizontal orientation while zeaxanthin does not. Contrary to common believe, lutein horizontal orientation is not related to the *ε*-ring rotation around the C6′-C7′ bond.

## Introduction

Xanthophylls (polar carotenoids) are natural pigments, synthesised almost entirely by plants and bacteria. They play important and diverse roles in living organisms, including animals, despite the fact that animals cannot synthesize xanthophylls and have to obtain them from foods. In brief, the biological functions of xanthophylls (XAN) can be divided more or less into those on the organism/tissue level and those on the molecular level. The former include photoprotection, antioxidation, energy transfer, disease prevention and cognitive function, while the latter include structural, modulatory and regulatory functions. XANs have been studied for almost 200 years and there is a rich literature on their biosynthesis, transport, localisation and functions etc. Examples of the more recent representative papers include (McNulty et al., 2007; Ballottari et al., 2013; Balevicius et al., 2017; Grudzinski et al., 2017; Mohn et al., 2017; Thomas and Johnson, 2018; Stringham et al., 2019; Demmig-Adams et al., 2020a; Demmig-Adams et al., 2020b; Johra et al., 2020; Seel et al., 2020; Widomska et al., 2020; Chia et al., 2021; Luchowski et al., 2021; Widomska et al., 2021).

**FIGURE 1:**
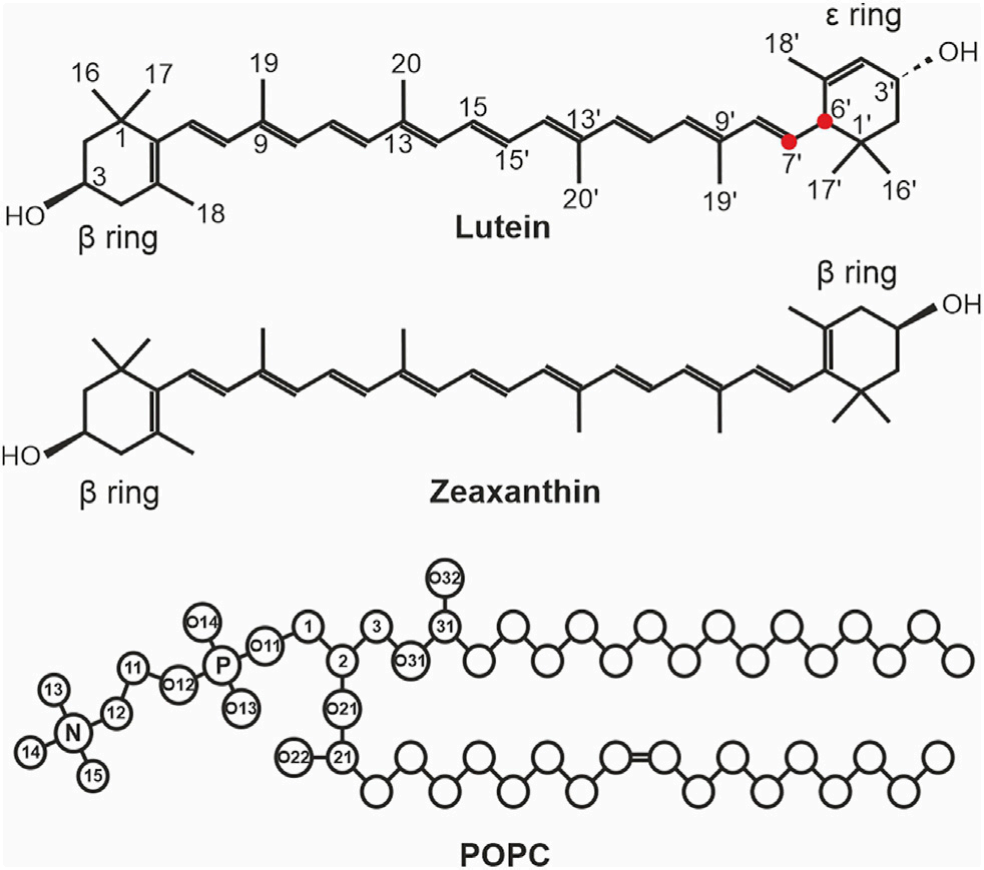
The chemical structures of lutein, zeaxanthin, and 3-palmitoyl-2-oleoyl-1-phosphatidyl-choline (POPC). The ionone rings and atoms that are used in the analyses are indicated in lutein only. The C6′ and C7′ atoms of lutein are indicated with *red* circles. The XAN atoms in the scheme are numbered according to the IUPAC convention (Nomenclature, I.C.o.t.N.o.O.C.a.t.I.-I.C.o.B., 1975). In POPC, the head group atoms are numbered in accordance with Sundaralingam (Sundaralingam, 1972). The numbers of the atoms of the acyl chains, the chemical symbols for carbon atoms, C, and the hydrogen atoms have been omitted except for the OH groups of lutein and zeaxanthin.

The most abundant XANs in humans are lutein and zeaxanthin (Figure 1). They preferentially locate in the retina where they account for 100% of the total retina carotenoid content (Whitehead et al., 2006) and in the brain where they account for 70% of the total brain carotenoid content (Johnson et al., 2013). In these organs, lutein and zeaxanthin play a mainly protective role.

Zeaxanthin and lutein (Figure 1) consist of a fairly rigid nonpolar polyene chain with two ionone rings at either end monohydroxylated at positions C3 and C3´. This structure promotes the transmembrane location of the molecules. Both zeaxanthin rings are identical (β-rings) and all its double bonds are conjugated (Figure 1). Lutein differs from zeaxanthin in the location of one double bond in one of the ionone rings (ε-ring). This bond is separated from the terminal double bond of the polyene chain by two single bonds (Figure 1). In effect, lutein has three chiral centres and a *3R, 3′R, 6′R* configuration, whereas zeaxanthin has two chiral centres and a *3R, 3′R* configuration. Moreover, the planes of the lutein *ε* and *ß* rings form an almost 90° angle with each other and the two hydroxyl groups are oppositely directed (cf. Figure 9 in Ref. (Makuch et al., 2019)). Thus, the lutein rings are not equivalent and not all of lutein double bonds are conjugated. This seemingly small difference in the structures of both molecules has significant biological repercussions summarised, e.g., in Ref. (Widomska et al., 2020). This structural difference was used to explain the sporadic horizontal location of lutein (but never of zeaxanthin) in lipid bilayers, observed in earlier experiments, e.g., (Gruszecki, 1999; Sujak et al., 2002; Tan et al., 2013). The reasoning applied there was that the two single bonds (C5′-C6′, and C6′-C7′, Figure 1) give the *ε*-ring relative freedom to rotate around the C6′-C7′ bond (Gruszecki, 1999; Sujak et al., 2002; McNulty et al., 2007). This rotation was assumed to enable both lutein rings to anchor in the same bilayer interface. A similar rotation was seemingly not possible for the *ß*-ring either of lutein or zeaxanthin, so zeaxanthin could locate in the bilayer only vertically to the bilayer surface. A more recent experimental study of Gruszecki’s group indicated that both xanthophylls located vertically in the bilayer (Grudzinski et al., 2017), although the results in Ref. (Elkholy et al., 2020) were interpreted under the assumption that there were two pools of orthogonally oriented lutein molecules in the bilayer. In partial agreement with experimental studies, computer modelling indicated that on a hundred nanosecond timescale, which is relatively short, lutein could locate horizontally below the bilayer interface, although its preferential orientation was transmembrane (Pasenkiewicz-Gierula et al., 2012; Makuch et al., 2019). A very recent publication of Luchowski et al. (2021) showed, using both experimental and computational methods, that the orientation of all-*trans* xanthophylls in the bilayer is transmembrane however, their *cis* isomers can locate parallel to the bilayer surface. *Trans*-*cis* photoisomerization of xanthophylls shortens their hydrophobic length and this enables them to reorient from the vertical to horizontal position. The horizontal position in the retina was shown to play a crucial protective role in the human eye. Thus, the issue of the xanthophyll orientation in the bilayer is of biological relevance.

The quantum mechanical calculations of Landrum et al. (2010) provided energy profiles for rotation around the C6-C7 and C6′-C7′ bonds of lutein (cf. Figure 4 of Ref. (Landrum et al., 2010) and Figure 1). Each profile has two energy minima representing two stable conformations of each torsion angle. The minima for rotation around the C6-C7 bond are very close to each other (–30°, +30°) and are separated by a relatively low barrier. So transitions between the two low energy states are quite likely. The minima for rotation around the C6′–C7′ bond (−50°, +130°) are separated by ∼180° and a relatively high barrier. So transitions between the two low energy states are much less likely.

In our previous computational studies, the process of lutein intercalation into the phosphatidylcholine (PC) bilayer was investigated in detail (Pasenkiewicz-Gierula et al., 2012; Makuch et al., 2019). Those studies showed that lutein inserts into the bilayer spontaneously and fast, within 20–100 ns. However, their main contribution was to demonstrate that insertion is not symmetric–lutein enters the bilayer predominantly with its *ß*-ring (Makuch et al., 2019). This asymmetric insertion of lutein into the membrane is strongly coupled with asymmetric binding of lutein by certain proteins (Horvath et al., 2016; Shyam et al., 2017), e.g., isomerohydrolase which catalyses the isomerization of lutein to meso-zeaxanthin; the *ε*-ring fits better to the ring-binding pockets of the protein than the *ß*-ring (Shyam et al., 2017).

In this study, more detailed issues are addressed; among them the rotation of the lutein *ß*-ring and *ε*-ring around the C6-C7 and the C6′-C7′ bond, respectively, in the bilayer, water and vacuum; the timescale of the free reorientation of lutein and zeaxanthin from the horizontal to the vertical orientation in the bilayer; the types and lifetimes of the interatomic interactions that stabilise a given orientation of the molecules in the bilayer and the corresponding conformations of the C6–C7 and C6′–C7′ bond torsions, and also the rotational mobility of the xanthophyll molecules in the bilayer.

## Materials and Methods

In this study, classical molecular dynamics (MD) simulation with atomic resolution was used to investigate the dynamic behaviour, orientation and intermolecular interactions of lutein and zeaxanthin in the PC bilayer.

### Simulation Systems Construction

**FIGURE 2:**
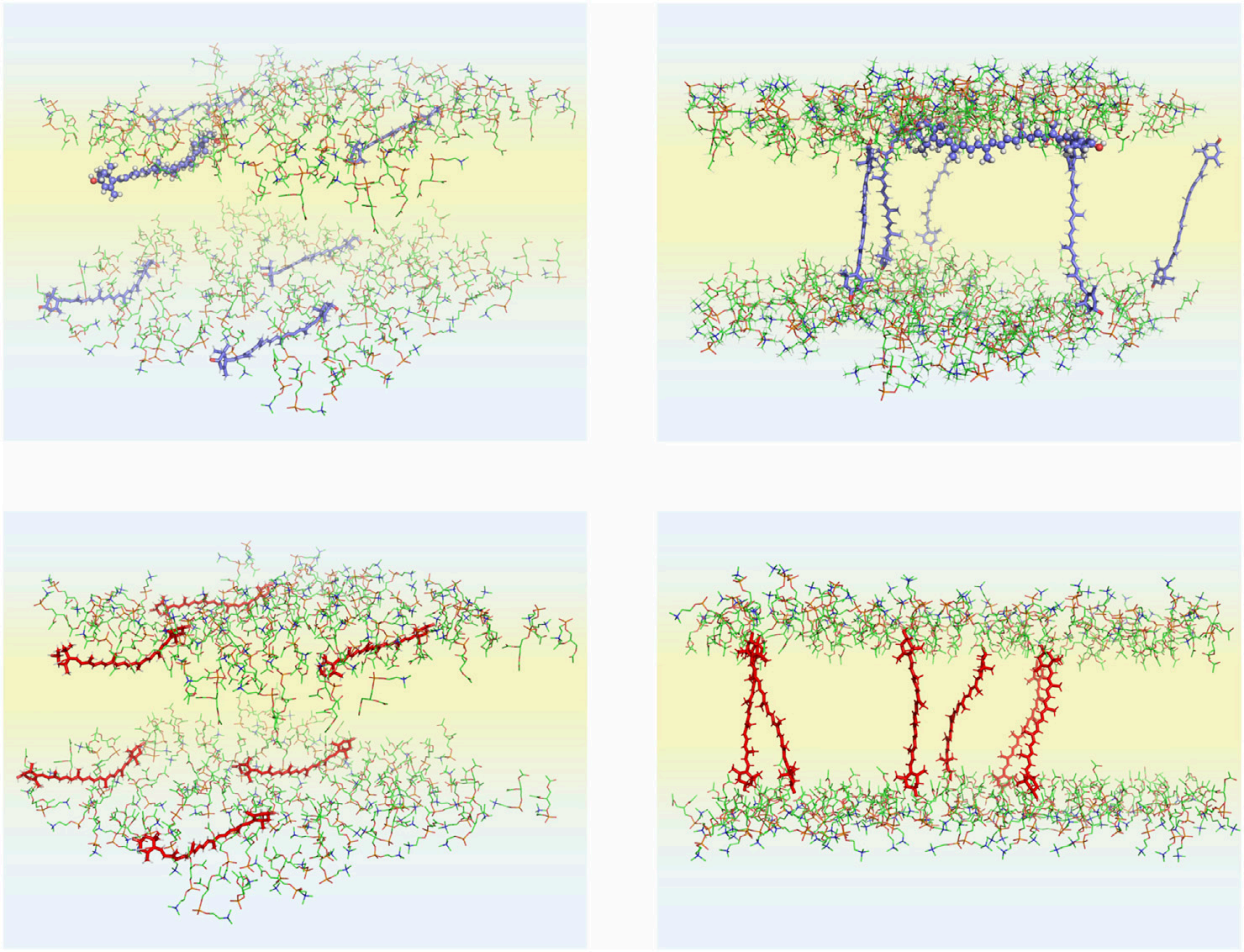
Initial **(left column)** and final **(right column;** after 1,100 ns of MD simulations) structures of the POPC-LUT_H **(upper row)** and POPC-ZEA_H **(lower row)** bilayers. Initially, six xanthophyll molecules are placed parallel to the bilayer surface. In the figures, only the POPC head groups and xanthophyll molecules are shown; the POPC acyl chains, water molecules and the PC head group hydrogen atoms are removed to better show the details of the systems. The atoms are represented in standard colours, except for the xanthophyll carbon atoms which for are *blue* for lutein *red* for zeaxanthin. Water is shaded *light blue* and the lipid nonpolar region is shaded *light yellow*. The lutein molecule (#6) that remained in the horizontal position during the whole simulation time of the POPC-LUT_H bilayer is presented in the ball-and-stick model, both in the initial and final states.

In this MD simulation study, several bilayer systems were constructed. Each bilayer consisted of 188 3-palmitoyl-2-oleoyl-1-phosphatidylcholine (POPC, Figure 1), six lutein or zeaxanthin and ∼8,500 H_2_O, molecules. To construct the systems, a POPC bilayer containing 200 POPC (100 in each leaflet) and 6,000 water molecules, built, equilibrated and validated in Ref. (Plesnar et al., 2012), was used. To construct the initial structures, the water molecules were removed from the POPC bilayer and twelve POPC molecules were substituted with six xanthophylls molecules. In the first system the lutein molecules were placed vertically (PC-LUT_V) to the bilayer surface, and in the second they were placed horizontally (PC-LUT_H) at the depth between the glycerol and the phosphate groups regions. The third and fourth systems contained zeaxanthin molecules oriented in the same way as in the first and second systems, PC-ZEA_V and PC-ZEA_H, respectively. Then, each system was rehydrated with over 40 water molecules per lipid. The initial and final (after 1.1 µs of MD simulation) structures of the PC-LUT_H and PC-ZEA_H systems are shown in Figure 2.

To compare the rotational freedom of the *ß* and *ε* rings of lutein as well as the two *ß* rings of zeaxanthin in different environments, i.e., in the bilayer, water and vacuum, accompanying systems were constructed. Two of them comprised a water box containing ∼4,000 water molecules and a single lutein or zeaxanthin molecule, the other two were single molecules in vacuum.

To elucidate how the behaviour of the C5′-C6′-C7′-C8′ torsion angle depends on its initial conformation, two additional systems were constructed. One of them comprised a lutein molecule with the initial conformation of the C5′-C6′-C7′-C8′ torsion angle of −50° (LUT-E50) in the water box, the other, in vacuum. −50° corresponds to the higher minimum in the energy profile for rotation around the lutein C6′–C7′ bond (cf. Figure 4A of Ref. (Landrum et al., 2010) and Figure 1). The initial −50° conformation of the torsion angle was imposed only in the latter two systems, which were termed constrained systems; in all other systems the conformation of none torsion was pre-set and they were termed unconstrained systems.

The initial structure of the *3R, 3′R, 6′R* lutein molecule with all torsion angles in the polyene chain in the *trans* conformation was created as described in Refs. (Pasenkiewicz-Gierula et al., 2012; Makuch et al., 2019). The structure of zeaxanthin, which has two chiral centres in the *3R, 3′R* configuration and two *ß*-rings, was based on that of lutein.

### Force Field and Simulation Parameters

For POPC, lutein and zeaxanthin, all-atom optimized potentials for liquid simulations (OPLS-AA) force field (Jorgensen et al., 1996) was used, and for water, the TIP3P model (Jorgensen et al., 1983) was used. To check the correctness of the OPLS-AA parameterisation of xanthophylls, the OPLS-AA profile for rotation about the lutein C6′-C7′ bond was computed and together with the flex angle (C3′-C6′-C7′) profile is shown in SupplementaryFigure S0 (Supporting Information, SI). The minima and the barriers in the OPLS-AA profile are very close to those in (Landrum et al., 2010) for the hydroxy-substituted *ε*-ring of lutein. As can be seen in Supplementary Figure S0, the value of the flex angle changes with the rotation of the *ε*-ring around the C6′-C7′ bond. But also, what is important, the shape of the rotational profile strongly depends on the initial value of the flex angle. Other results that validate the OPLS-AA parameterisation are further down in the text.

MD simulations of the bilayer and water systems were carried out in the *NPT* ensemble at a physiological temperature of 310 K (37°C) and pressure of 1 atm using the GROMACS software package (Hess et al., 2008). The temperatures of the solute and solvent were controlled independently using the Nosé-Hoover method (Nose, 1984; Hoover, 1985) with a relaxation time of 0.6 ps. The pressure was controlled anisotropically using the Parrinello-Rahman method (Parrinello and Rahman, 1981) with a relaxation time of 1.0 ps. MD simulations of xanthophyll molecules in vacuum were carried out in the *NVT* ensemble. The linear constraint solver (LINCS) algorithm (Hess et al., 1997) was used to preserve the length of any covalent bond with a hydrogen atom, and the time step was set to 2 fs. The van der Waals interactions were cut off at 1.2 nm. The long-range electrostatic interactions were evaluated using the particle-mesh Ewald summation method with a *ß*-spline interpolation order of 5 and direct sum tolerance of 10^−6^ (Essmann et al., 1995). For the real space, a cut-off of 1.2 nm, three-dimensional periodic boundary conditions, and the minimum image convention were used (Essmann et al., 1995). The list of nonbonded pairs was updated every five time steps.

Each of the bilayer systems was MD simulated for 1.1 µs and each system containing a single XAN molecule was MD simulated for 100 ns which is long enough to sample possible configurations of a single ∼100-atom xanthophyll molecule. The atomic coordinates in each system were recorded every 1 ps. During MD simulations, conformations of the polyene chain torsion angles as well as separation of the xanthophyll molecules in the systems were monitored. The initial *trans* conformation of all torsion angles was preserved and in none of the bilayers the xanthophyll molecules aggregated to form temporary stable dimers or larger aggregates, although, from time to time, some of their atoms made relatively close transient contacts.

## Results and Discussion

### Hopping Rotation of the ε-ring and *ß*-ring

#### Unconstrained systems

**FIGURE 3:**
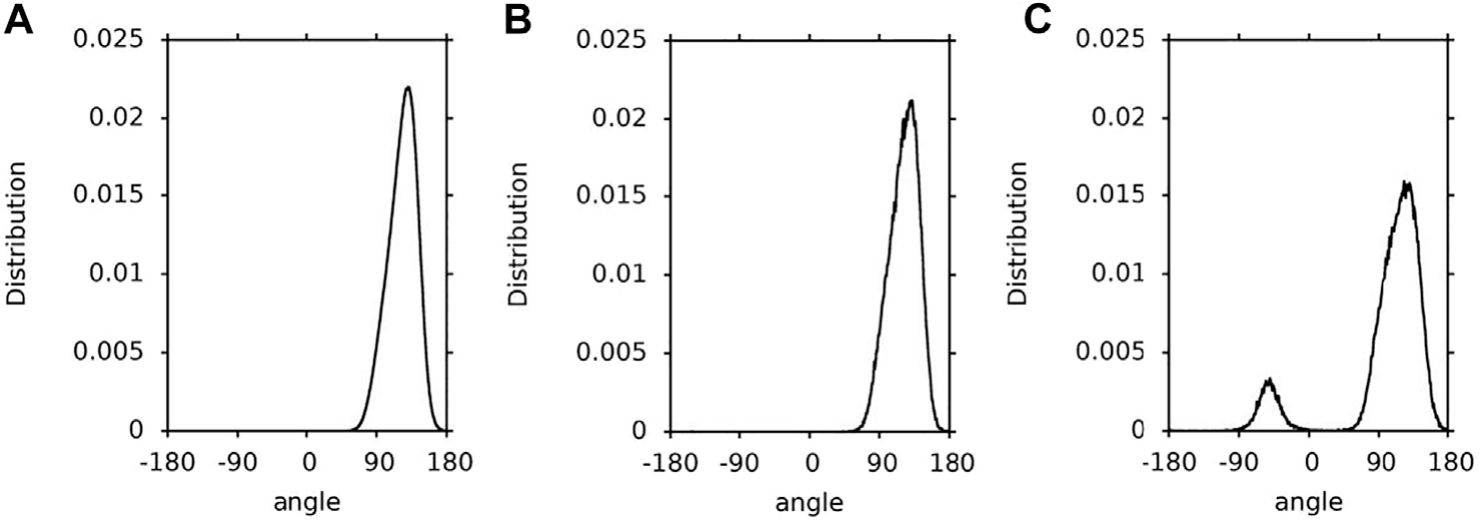
Population distributions of lutein C5′-C6′-C7′-C8′ torsions angles (ε-ring torsion). **(A)** in the PC-LUT bilayer; **(B)** in water; **(C)** in vacuum.

**FIGURE 4:**
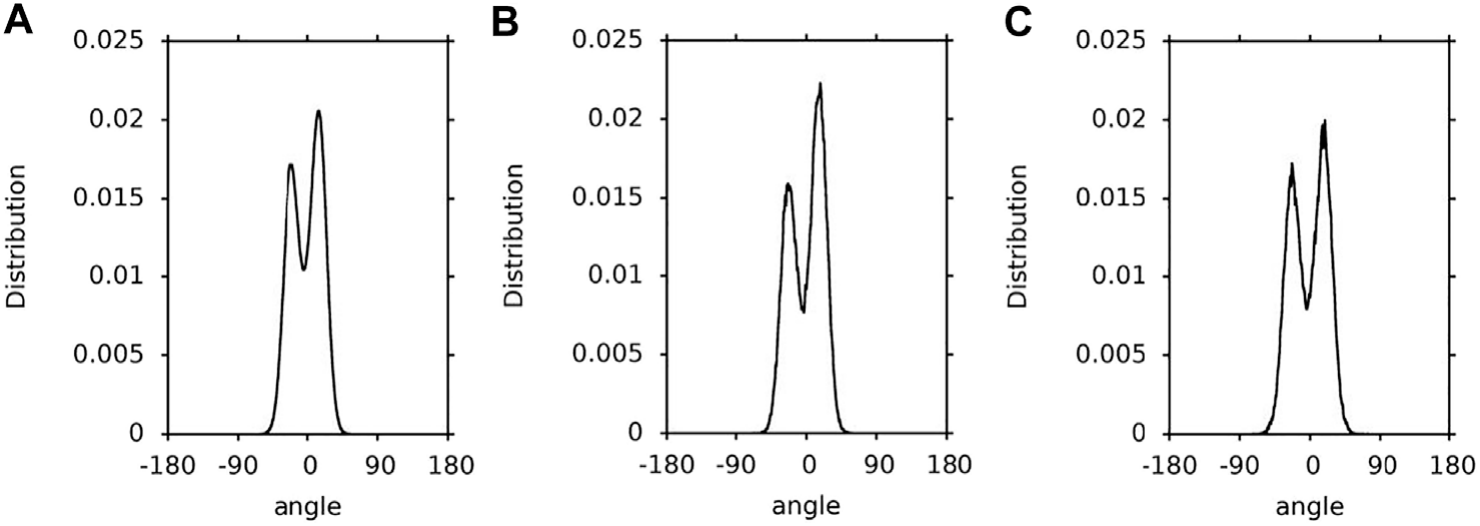
Population distributions of the lutein C5-C6-C7-C8 torsion angles (β-ring torsion). **(A)** in the PC-LUT bilayer; **(B)** in water; **(C)** in vacuum.

**FIGURE 5:**
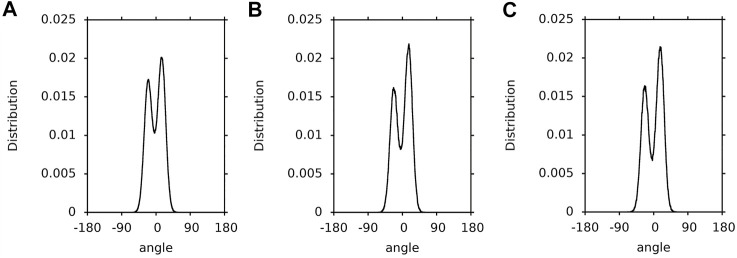
Population distributions of the zeaxanthin *ß*-ring torsions. **(A)** in the PC-ZEA bilayer; **(B)** in water; **(C)** in vacuum.

In this paper, a hopping rotation describes the rotational motion of a XAN ring. This rotation results from −50°↔130° isomerisation of the C5′-C6′-C7′-C8′ torsion angle (*ε*-ring) and −30°↔30° isomerization of the C5-C6-C7-C8 torsion angle (*β*-ring). For these angles the energy profiles for rotation presented in Figures 4A,B of Ref. (Landrum et al., 2010) and in Supplementary Figure S0 have minima. The population distributions of the C5′-C6′-C7′-C8′ and C5-C6-C7-C8 torsions angles (called *ε*-ring and *ß*-ring torsion, respectively, for short) of lutein and zeaxanthin (the *ß*-rings of zeaxanthin are indistinguishable) in the bilayer, water and vacuum, and which were obtained in this study, are shown in Figures 3–5, respectively. The population of the *ε*-ring torsions of lutein in vacuum (∼1:4) reflects the energy profile for rotation around the C6′-C7′ bond (Figure 3C) of the hydroxy-substituted lutein *ε*-ring calculated at the B3LYP/6-31G* level of theory in Ref. (Landrum et al., 2010) and the OPLS-AA profile in Supplementary Figure S0. Both profiles have two minima differing by ∼1 kcal/mol in depth and an energy barrier between them of ∼6 kcal/mol. In contrast, the populations of the *ε*-ring torsions of lutein in the bilayer and in water (Figures 3A,B) seem to be in accord with the energy profile of the hydroxy-unsubstituted lutein *ε*-ring calculated in Ref. (Landrum et al., 2010) (cf. Figure 4A of Ref. (Landrum et al., 2010)), where the energy barrier between the two minima is higher and the difference in the depths of the minima is larger.

1.1-µs time profiles of the *ε*-ring torsion of the six lutein molecules in the PC-LUT_H bilayer are shown in Supplementary Figure S1 and in the PC-LUT_V bilayer in Supplementary Figure S2; 100-ns time profiles of the *ε*-ring torsion of one molecule in water and in vacuum are shown in Supplementary Figure S3. The profiles are consistent with the distributions of the torsion angles in Figure 3. In all environments, the *ε*-ring torsion oscillates between 100 and 150°, and also between −20° and −70° for lutein in vacuum, which corresponds to fairly broad distributions of these torsion angles (Figure 3). For the lutein molecules in the bilayers, with the exception of two cases, there are no transitions between the two low energy conformations (130 and −50°) of the *ε*-ring torsion. One of the transitions occurs in the PC-LUT_H bilayer at ∼1,010 ns of MD simulation and lasts for ∼10 ns, then the conformation returns to the previous one (Supplementary Figure S1). The other occurs in the PC-LUT_V bilayer at ∼650 ns of MD simulation and lasts for less than 200 ps. It should be emphasized here that there are altogether twelve lutein molecules in two bilayers and in each bilayer their behaviour is MD simulated for 1.1-µs. Thus, on the basis of these statistics one can conclude that there is essentially no hopping rotation of the *ε*-ring around the C6′-C7’ bond of lutein in the bilayer (and in water), whereas there is a frequent hopping rotation of the ring in vacuum. However, in all systems, i.e., in the bilayer, water and vacuum, the *ε*-ring torsions fluctuate significantly (Supplementary Figures S1, S2, S3).

To find an explanation for why the *ε*-ring torsion of lutein populates different conformations in different environments, the interactions between the *ε*-ring OH group and the environment were checked. In the water box, this group forms, on average, ∼2.3 hydrogen bonds (H-bond) with water (Table 1). In the bilayer, it makes a similar number of intermolecular interactions with PC polar groups and water (cf. *Interactions Stabilising XAN Orientation in the Bilayer*, Tables 1,2). In vacuum, none of these interactions takes place. The interactions of the *ε*-ring OH group in water and in the bilayer hinder isomerisation between two minimum energy conformations of 130° (lower energy) and −50° (higher energy) because it requires not only that the energy barrier be overcome, whose height calculated in vacuum is ∼6 kcal/mol (Landrum et al., 2010), but also that interactions between water molecules and PC head groups be broken.

**TABLE 1:** Numbers of H-bonds of XAN-OH.

#H-bonds	Lutein E	Lutein B	Zeaxanthin B
Op	0.05 ± 0.22 H 0.14 ± 0.15 V	0.02 ± 0.16 H 0.11 ± 0.14 V	0.11 ± 0.09
O22	0.08 ± 0.27 H 0.11 ± 0.12 V	0.16 ± 0.37 H 0.12 ± 0.13 V	0.14 ± 0.11
O32	0.23 ± 0.42 H 0.07 ± 0.10 V	0.11 ± 0.31 H 0.06 ± 0.10 V	0.06 ± 0.07
All	0.35 ± 0.48 H 0.32 ± 0.19 V	0.29 ± 0.46 H 0.30 ± 0.18 V	0.31 ± 0.14
Water	0.68 ± 0.64 H 1.21 ± 0.30 V	0.96 ± 0.75 H 1.38 ± 0.31 V	1.33 ± 0.20
H_2_O···OH	E130: 2.33 ± 0.1 E50: 2.04 ± 0.1	2.32 ± 0.1	2.31 ± 0.1

Time and ensemble average numbers of H-bonds (#H-bonds) of a XAN-OH group with a specified PC oxygen atom, with all PC oxygen atoms (All) and with water molecules (Water), in the PC-LUT and PC-ZEA bilayers as well as with water molecules in the water box (H_2_O···OH), per OH and per ps. Op, O22 and O32 are PC oxygen atoms (cf. Figure 1); E specifies the ε ring, E130 and E50 label two conformations of the ε ring torsion, 130° and –50°, respectively, B specifies the *ß*-ring. H and V label the final position of the lutein molecule in the bilayer–H stands for horizontal, V for vertical.

**TABLE 2:** Time and ensemble average numbers of intermolecular interactions between a XAN-OH group and PC, per OH and per ps.

#Water-bridges	Lutein E	Lutein B	Zeaxanthin B
Op	0.14 ± 0.36 H 0.32 ± 0.21 V	0.13 ± 0.38 H 0.35 ± 0.22 V	0.34 ± 0.16
O22	0.06 ± 0.24 H 0.17 ± 0.15 V	0.14 ± 0.36 H 0.21 ± 0.16 V	0.21 ± 0.12
O32	0.17 ± 0.39 H 0.17 ± 0.14 V	0.24 ± 0.47 H 0.19 ± 0.17 V	0.19 ± 0.11
All	0.37 ± 0.59 H 0.66 ± 0.30 V	0.51 ± 0.66 H 0.74 ± 0.31 V	0.75 ± 0.22
#Charge-pairs	0.22 ± 0.66 H 0.70 ± 0.36 V	0.46 ± 0.74 H 0.57 ± 0.33 V	0.73 ± 0.22
Head group	0.94 ± 1.01 H 1.68 ± 0.51 V	1.26 ± 1.09 H 1.61 ± 0.49 V	1.79 ± 0.34

Water bridges (#Water-bridges) with a specified PC oxygen atom and with all PC oxygen atoms (ALL); charge pairs (#Charge-pairs) with the PC choline group; all types of OH-PC interactions (Head group), in the PC-LUT and PC-ZEA bilayers. Op, O22 and O32 are PC oxygen atoms (cf. Figure 1); E specifies the *ε* ring, B specifies the *ß*-ring. H and V label the final position of the lutein molecule in the bilayer–H stands for horizontal, V for vertical.

The population distributions (∼1:2) of the *ß*-ring torsions are very similar for lutein in the bilayer, water and vacuum (Figure 4). They reflect the energy profiles for rotation around the C6-C7 bond (Figure 1) of hydroxy-substituted lutein *ß*-ring calculated in Ref. (Landrum et al., 2010) (cf. Figure 4B of Ref. (Landrum et al., 2010)). Each of the profiles has two minima that do not differ greatly in depth and are close to each other, and a barrier between them of ∼4 kcal/mol. The effect of the environment on the conformational freedom of the *ß*-ring torsion is much smaller than that on the *ε*-ring torsion, even though the numbers of intermolecular interactions made by the *ß*-ring and *ε*-ring OH groups are similar (cf. *Interactions Stabilising XAN Orientation in the Bilayer*, Tables 1, 2). This is most likely because the energy barrier for rotation around the C6-C7 bond is low enough (∼4 kcal/mol (Landrum et al., 2010)) and the minima are close to each other and of a similar depth, so isomerisation does not require intermolecular interactions to be broken and is not hindered.

1.1-µs time profiles of the *ß*-ring torsions of the six lutein molecules in the PC-LUT_H bilayer are shown in Supplementary Figure S1 and for the PC-LUT_V bilayer in Supplementary Figure S2, and 100-ns time profiles of the *ß*-ring torsion of one molecule in water and vacuum are shown in Supplementary Figure S4. The profiles are consistent with the distributions of the torsion angles in Figure 4. The *ß*-ring torsion not only fluctuates but it very frequently transits between the two minimum energy states; particularly frequent are transitions in water and vacuum. It should be emphasised that the two minima are very close to each other (30° and −30°), thus, in each environment, the torsion can easily adopt any of the two conformations corresponding to the two neighbouring low energy states. Nevertheless, the lower energy conformation of 30° is by a factor of two more populated than the higher energy one of −30°. Thus, the *ß*-ring, in contrast to the *ε*-ring which in practice does not undergo hopping rotation, frequently rotates around the lutein C6-C7 bond in the bilayer, water and vacuum. This rotation, however, is of a very limited angular amplitude.

Supplementary Figures S1, S2 indicate that conformational behaviour of the *ε*-ring and *ß*-ring torsions does not depend on the orientation of lutein in the bilayer (horizontal or vertical).

Not surprisingly, the population distributions of the *ß*-ring torsions of zeaxanthin in the bilayer, water and vacuum (Figure 5) are very similar to those for the *ß*-ring torsions of lutein (Figure 4) and similar to one another.

Furthermore, the 1.1-µs time profiles of the zeaxanthin *ß*-ring torsions in the PC-ZEA_H and PC-ZEA_V bilayers (Supplementary Figure S5), as well as the 100-ns time profiles of the *ß*-ring torsion in water and vacuum (Supplementary Figure S6), are very similar to those of the lutein *ß*-ring torsions (Supplementary Figures S1, S2, S4), thus the behaviour of the *ß*-ring of zeaxanthin and of lutein is actually the same.

#### Constrained systems

**FIGURE 6:**
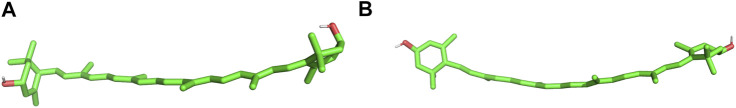
3D images of lutein in water. The ε-ring (on the left) torsion in the **(A)** –50° and **(B)** 130° conformations. In both conformations the ε-rings are almost perpendicular to the polyene chain and, at the same time, their planes are mutually perpendicular.

The constrained systems are analysed to elucidate the conformational behaviour of the lutein *ε*-ring torsion when its initial conformation is pre-set to −50°. The 3D structures of the lutein molecule in two minimum energy conformations (−50° and 130°) are shown in Figure 6.

The 100-ns time profiles as well as population distributions of the *ε*-ring torsions pre-set to −50° of a lutein molecule (LUT-E50) in water and in vacuum are shown in Supplementary Figure S7. Supplementary Figure S7 indicates that in water the *ε*-ring torsion undergoes a single transition to the lower energy stable conformation of 130° at ∼10 ns of MD simulation and stays in this conformation for the remaining simulation time, i.e., till 100 ns. In vacuum, the behaviour of the *ε*-ring torsion of LUT-E50 is very similar to that when the initial conformation is 130° (Supplementary Figures S3B, S7B), i.e., there is a frequent isomerisation of the *ε*-ring torsion between the –50° and 130° conformations. In both cases, however, the 130° conformation is significantly more populated than the –50°, in accord with the energy profile for rotation around the C6′-C7’ bond shown in Ref. (Landrum et al., 2010) and that in Supplementary Figure S0. Thus, the conformational behaviour of the *ε*-ring torsion does not depend on which of the minimum energy conformations is the initial one.

Even though the OH group of the *ε*-ring of LUT-E50 interacts with water in the water box (Table 1), the transition from −50° to 130° occurs. This single transition is possible because for conformation of −50°, the number of H-bonds between *ε*-ring and water is somewhat smaller than for 130° (Table 1). Besides, the minimum at −50° is less deep that at 130°, so the transition to 130° has to overcome a relatively lower barrier than in the case of the reverse transition, which, in general, does not take place (Supplementary Figure S1).

### Preferred Orientation of XAN in the Bilayer

**FIGURE 7:**
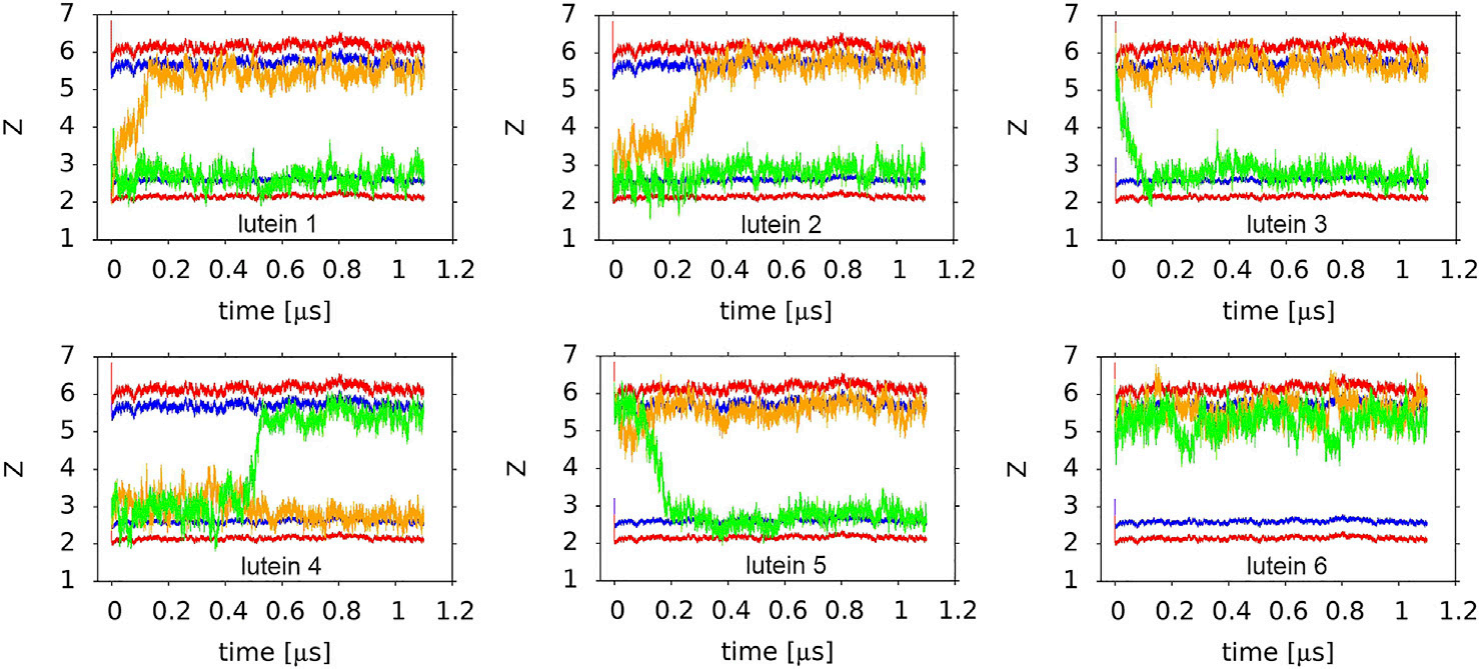
Time profiles of the orientations of six lutein molecules in the PC-LUT_H bilayer. The *orange* and *green* lines show the trajectories of the centre-of-masses of the *ß* (*orange*) and ε (*green*) rings along the *z*-axis. Two horizontal *red* and *blue* lines indicate the average vertical location of the POPC P and glycerol O atoms in each of the bilayer leaflets. The order of the plots corresponds to the lutein molecule numbering (#4 is the first and #6 is the last in the lower row).

Four basic bilayers used in this study contained six xanthophyll molecules each, i.e., 24 in total. Initially, twelve of them were placed vertically and twelve horizontally to the bilayer surface. The time courses of the orientations of the six lutein molecules in the PC-LUT_H bilayer during 1.1-µs MD simulation are shown in Figure 7 and Supplementary Film SF1, and those of the six zeaxanthin molecules in the PC-ZEA_H bilayer are shown in Supplementary Figure S8 and Supplementary Film SF2. As can be seen in Figures 2,7 and Supplementary Figure S8 and films, in eleven cases, the horizontally placed molecules change their positions to vertical in a free rotation. Only one lutein molecule remains in the horizontal position during the whole simulation time of 1.1 µs. In contrast, none of the vertically placed molecules in the PC-LUT_V (Supplementary Figure S9) and PC-ZEA_V bilayers changes its orientation to horizontal.

Reorientations of lutein and zeaxanthin molecules from the horizontal to vertical position in the PC-LUT_H and PC-ZEA_H bilayers, respectively, take place over a similar time range, between tens and hundreds of ns (the longest reorientation time for both types of molecules is ∼500 ns) (Figure 7 and Supplementary Figure S8).

### Interactions Stabilising XAN Orientation in the Bilayer

#### Polar interactions

Both lutein and zeaxanthin have two polar OH groups (XAN-OH), which are both H-bond donors and acceptors. Thus, these groups can interact with water and polar groups of PC. To establish whether such interactions take place, radial distribution functions (RDF) were calculated and for lutein they are shown in Supplementary Figures S10–S12. The authors also checked which atoms and groups of PC interact with XAN-OH. The RDFs in Supplementary Figure S10 indicate that LUT-OH forms H-bonds with water molecules that hydrate the bilayer. The RDFs in Supplementary Figure S11 indicate that it forms H-bonds with non-esterified phosphate, O13 and O14 (collectively named Op), and carbonyl, O22 and O32 (collectively named Oc), oxygen atoms of PC but not with the esterified phosphate, O11 and O12 (collectively named Oe), and glycerol O21 and O31 (collectively named Og) (cf. Figure 1) oxygen atoms of PC. The RDFs in Supplementary Figure S12 indicate that the LUT-OH also interacts with the PC choline (N-CH_3_) group (forms charge pairs, (Pasenkiewicz-Gierula et al., 1999)). RDFs for the zeaxanthin OH groups are similar to those of lutein (not shown).

The XAN-PC interactions can be direct, via H-bonds and charge pairs (Pasenkiewicz-Gierula et al., 1999; Szczelina et al., 2020), and indirect, *via* a water molecule that is simultaneously H-bonded to a XAN-OH and one of the oxygen atoms of PC (Pasenkiewicz-Gierula et al., 1997; Szczelina et al., 2020). The numbers of the XAN-OH···PC and XAN-OH···H_2_O H-bonds, XAN-OH—PC water bridges and charge pairs were calculated every ps. Their time and ensemble average values in the PC-LUT and PC-ZEA bilayers per OH group, are given in Tables 1, 2, respectively. In Table 1, the XAN-OH···H_2_O H-bonds/OH of lutein and zeaxanthin in a water box are also given.

On the basis of the entries in Tables 1, 2 it is difficult to infer why one of the six lutein molecules in the PC-LUT_H bilayer remained in the horizontal orientation, whereas the other five rotated spontaneously to the transmembrane position. The reverse rotation from the vertical to the horizontal orientation never happened during 1.1-µs MD simulations either in the case of the six lutein and six zeaxanthin molecules in the PC-LUT_V and PC-ZEA_V bilayers, respectively, or those eleven which had rotated in the PC-LUT_H and PC-ZEA_H bilayers. In all bilayers, each XAN-OH group makes relatively few interactions with PC. Moreover, as there is only one horizontal lutein, the numbers of its interactions with specific PC atoms and groups (Tables 1, 2) bear large errors (standard deviations). Nevertheless, some difference in polar XAN-PC interactions between the molecules in mutually orthogonal orientations can be pointed out. Both rings of the horizontal lutein form a negligible number of H-bonds with Op, but the numbers of H-bonds with O32 and water bridges with Op are significantly larger, although absolutely small, of ∼0.2/OH and ∼0.1/OH, respectively, (Tables 1, 2). The numbers of H-bonds of the rings of the vertical lutein with Op and O32 are almost the reverse of those for the horizontal molecule, but the total numbers of OH···PC H-bonds are similar for the molecules in both orientations (Table 1) and are similar to those obtained in a computational study of Grudzinski et al. (2017). The largest differences in the XAN-PC polar interactions between the horizontally and vertically oriented lutein molecules are in the numbers of water bridges and charge pairs, particularly for the *ε*-ring (Table 2). Consistently with the results for XAN-OH···Op H-bonds, the average numbers of XAN-OH···H_2_O H-bonds made by each of the ionone rings of the horizontal lutein of ∼1 are smaller than those of a vertical one of ∼1.3. These indicate that the rings of the horizontal lutein are buried deeper in the bilayer nonpolar core than those of the vertical ones. The total average number of H-bonds, water bridges and charge pairs between a XAN ring and a PC head group, is ∼1 for each ring of the horizontal lutein and ∼1.6 (Table 2) for each ring of the vertical lutein. The numbers of the particular and total interactions of the *ß*-rings of zeaxanthin (only vertically oriented) with a PC head group and water molecules are very similar to those of the *ß*-ring of lutein in the vertical orientation (Tables 1, 2). Thus, anchoring in the bilayer interface is stronger for the vertically positioned XAN molecules than the horizontally positioned ones. This is one of the reasons why XAN molecules in the bilayer do not change their orientation from vertical to horizontal, whereas the reversed change is likely.

A comparison of the numbers of the polar interactions of the *ß* and *ε* rings with PC and the bilayer water indicates that for the horizontal lutein (#6, cf. *Orientation of the XAN Polyene Chain in the Bilayer*), the *ε*-ring makes fewer such interactions than the *ß*-ring, whereas for the vertical lutein, the numbers are almost the same. This indicates that the *ß*-ring of the horizontal lutein is closer to the bilayer water phase than the *ε*-ring, and this is in fact evident in Figure 7. However, for both horizontal and vertical lutein molecules, differences in the numbers of the interactions between the *ε* and *ß* rings are small and much smaller than the associated errors. Besides, it should be remembered that here the behaviour of one horizontal molecule (#6, cf. *Orientation of the XAN Polyene Chain in the Bilayer*) is compared with that of six vertical molecules.

#### Nonpolar interactions

**FIGURE 8:**
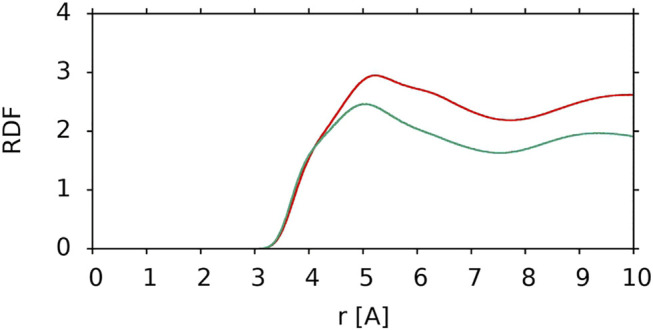
C-C RDF of the lutein polyene chain carbon atoms relative to PC acyl chain carbon atoms for a vertical lutein in the PC-LUT_V bilayer (*red*) and for the horizontal lutein in the PC-LUT_H bilayer (*green*). RDFs are cut at 10 Å.

To compare the interactions of the nonpolar polyene chain of lutein in two different orientations with the PC acyl chains, the RDFs of the polyene chain carbon atom relative to the PC acyl chain carbon atoms were calculated (C-C RDF). The C-C RDFs are compared in Figure 8. It is evident that when lutein is in the vertical position the nonpolar interactions between the lutein and PC chains are more favourable than when it is in the horizontal position. This is another reason why XAN molecules in the bilayer do not change their orientation from vertical to horizontal.

#### Orientation of the XAN Polyene Chain in the Bilayer

**FIGURE 9:**
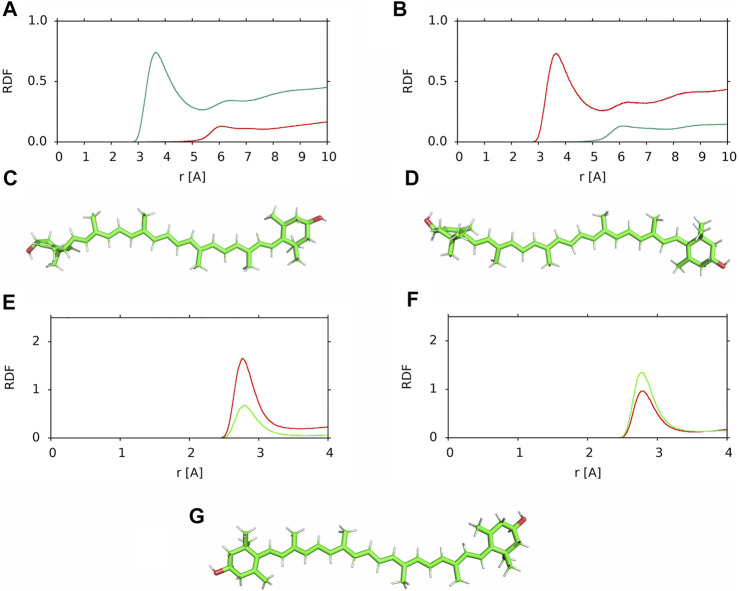
**(A,B)** RDF of water molecules relative to the MET groups of the (*red*) *ß*-ring and (*green*) *ε*-ring halves of the polyene chain in the PC-LUT_H bilayer **(A)** lutein #6, **(B)** lutein #4; **(C,D)** positions relative to the “upper” bilayer leaflet of the horizontally oriented lutein molecules in the PC-LUT_H bilayer **(C)** lutein #6, **(D)** lutein #4; **(E,F)** RDF of water molecules relative to the OH groups of the **(E)** *ß*-ring and **(F)** *ε*-ring in the PC-LUT_H bilayer (*red*) lutein #6 and (*green*) lutein #4; **(G)** position of zeaxanthin #1 in the horizontal position in the PC-ZEA_H bilayer. RDFs are cut at 10 or 4 Å.

The horizontal and vertical positions of a XAN molecule in the bilayer refer to the location of its long molecular axis relative to the bilayer surface. Here, the orientation of the polyene chain plane of a XAN molecule relative to the bilayer surface is checked. To this end, the RDFs of water molecules relative to the methyl groups (CH_3_, MET) of the *ß*-ring and *ε*-ring halves of the polyene chain were calculated for two horizontally positioned lutein molecules, #6, which remained in the horizontal position during the whole simulation time and #4 before it rotated to the vertical position at ∼500 ns of MD simulations; the RDFs are shown in Figure 9. The maxima and their positions in the RDFs for the MET groups of the *ε*-ring half of #6 (Figure 9A) and the *ß*-ring half of #4 (Figure 9B) indicate that they interact with water molecules which form a clathrate-like structure around them (Markiewicz et al., 2021), whereas the MET groups of the *ß*-ring half of #6 and the *ε*-ring half of #4 do not interact with water. The orientations of one half of the polyene chain of #6 towards the water phase and of the other towards the bilayer core are confirmed by the RDFs of the MET groups relative to the carbon atoms of the PC acyl chains in the PC-LUT_H bilayer (Supplementary Figure S14B). The results indicate that the plane of the polyene chain of the horizontally positioned lutein molecules is perpendicular to the bilayer surface and the MET groups of each half of the chain are oriented in the opposite directions along the bilayer normal (Figures 9C,D).

The orientation of the polyene chain relative to the bilayer surface was also checked for the zeaxanthin molecule #1 in the PC-ZEA_H bilayer. This molecule rotated from the horizontal to the vertical position at ∼500 ns of MD simulations (cf. Supplementary Figure S8). As in the case of the lutein #4 and #6, the plane of the polyene chain of horizontally positioned zeaxanthin #1 is perpendicular to the bilayer surface (Figure 9G).

Closer inspection of the positions of the XAN molecules positioned horizontally in the POPC bilayers (Supplementary Films SF1, SF2) indicates that during the initial 10 ns of MD simulations the plane of their polyene chains assumes a perpendicular orientation relative to the bilayer surface. Thus, this orientation of the polyene chain in the bilayer interfacial region is universal.

For horizontal lutein, the perpendicular orientation of the polyene chain has some consequences, as depending on the orientation of the MET groups, the OH group of the *ß*-ring is either oriented towards the water or the bilayer core phases (Figures 9C,D). In contrast, the orientation has little effect on the position of the OH group of the *ε*-ring in the bilayer, and therefore, on its interactions with the bilayer water as well. This can be seen in Figures 9E,F, where the RDFs of the water molecules relative to the *ß*-ring and *ε*-ring are shown—for both orientations the maxima of the RDFs for the ε-ring in #4 and #6 are similar, whereas that for the *ß*-ring of #6 is evidently higher than that for #4.

For horizontal zeaxanthin, the upward or downward orientation of the MET groups has no consequences because both zeaxanthin rings are *ß* and the OH group of one is oriented towards the water phase, while the other is oriented towards the bilayer core (Figure 9G). Thus zeaxanthin, compared to lutein, is much more predisposed to rotate across the bilayer as one of its *ß*-rings is always less anchored in the bilayer interface than the other and less than the lutein *ε*-ring (Figures 9E,F). This conclusion gets strong support from the result obtained in Ref. (Luchowski et al., 2021) that in *cis*-zeaxanthin both OH groups are on the same side of the molecule and this enables the molecule to ‘resides next to the membrane-water interface’ with both OH groups anchored in the same bilayer interface.

One would expect that when the OH group of a lutein (or zeaxanthin) *ß*-ring is oriented towards the bilayer core (*β*-down, Figure 9D), then most likely this ring drags its end of the molecule across the bilayer core to settle the OH group in a more favourable polar environment of the other leaflet interface, and when the OH group of a *ß*-ring is oriented towards the water phase (*β*-up, Figure 9C), it is less likely that the *ß*-ring will initiate rotation. As Figure 9F indicates, anchoring of the *ε*-ring OH group in the interface is almost the same for both *ß*-up and *ß*-down positions of lutein. But, even though in all six cases of zeaxanthin and two cases of lutein (#1 and #2) of the cross-bilayer rotation, the former was the case and in the two cases of lutein (#3 and #5) the latter was the case, the above expectation is nevertheless not a rule, as the example in Figure 7 of lutein #4 with the down *ß*-ring indicates. There, the *ε*-ring, and not the *ß*-ring end crosses the bilayer core.

### Rotation of XAN About the Long Axis

**FIGURE 10:**
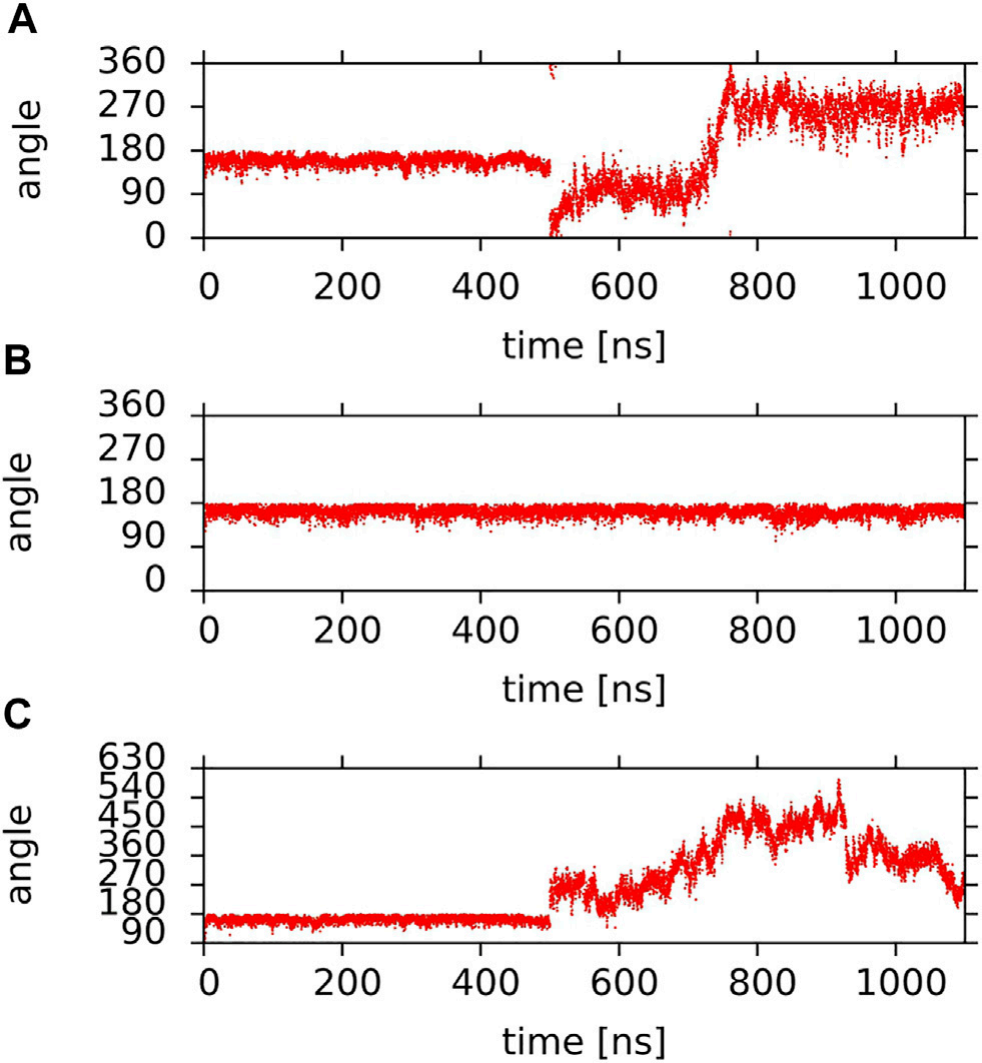
Time profiles of the θ angle of **(A,B)** lutein molecules **(A)** #4; **(B)** #6 in the PC-LUT_H bilayer; **(C)** zeaxanthin molecule #1 in the PC-ZEA_H bilayer (the *y*-axis scale was extended to avoid plot discontinuities due to jumps to 0° when 360° is approached). Lutein molecule #4 and zeaxanthin molecule #1 rotated to the vertical position at ∼500 ns of MD simulation (cf. Figure 7 and Supplementary Figure S9) and lutein molecule #6 remained in the horizontal position during the whole simulation time.

Here rotational freedom around the long axis of the lutein molecules in the PC-LUT_V bilayer as well as of lutein #4 and #6 in the PC-LUT_H bilayer and zeaxanthin #1 in the PC-ZEA_H bilayer, is assessed. The rotation is monitored as changes of the angle θ. For the vertically oriented molecules, θ is measured between the bond linking the MET group and C13 atom of the polyene chain, to which MET is attached (MET-C13 bond, Figure 1), and the *x*-axis in the *x-y*-plane; for the horizontally oriented molecules it is measured between the MET-C13 bond and the *z*-axis in a vertical plane, and recorded every 100 ps. The time profiles of the angles for the six lutein molecules in the PC-LUT_V bilayer are shown in Supplementary Figure S15, for lutein #4 and #6 and zeaxanthin #1 are shown in Figure 10. Whereas the vertically oriented molecules (Supplementary Figure S15) make at least one full 360° turn during the simulation time, the rotation around the long axis of the lutein molecules trapped in the horizontal positions is virtually blocked (Figure 10); the rotational freedom is restored once the entrapment is released (Figure 10A). The results in Figure 10 are consistent with those in Figure 9, which shows that MET groups on one half of the horizontally positioned XAN molecule faces only the water phase, while of the other half, only the bilayer core.

### Lifetime of XAN Polar Interactions in the Bilayer

The stability of the XAN orientation in the bilayer depends not only on the number of polar interactions of its OH groups with PC and water, given in Tables 1, 2, but also on their individual duration and continuity. An average duration of the interaction can be estimated by calculating the characteristic decay time using a method analogical to that described in Ref. (Róg et al., 2009). In short, at an arbitrarily chosen time point of the MD trajectory after equilibration, the number of specific interatomic interactions (H-bonds, water bridges or charge pairs) is established (initial number of interactions) and changes in the number over time, during a specified lag time (in this case 5 ns), are recorded. The initially established number decreases due to breaks in the interaction, but if the interaction is re-established at a later time, it is counted again. The procedure is repeated for several initial times and the results are averaged. This way, decay curves are obtained. If a decay curve is a smooth exponential function then the characteristic decay time can be obtained in the curve-fitting procedure.

**FIGURE 11:**
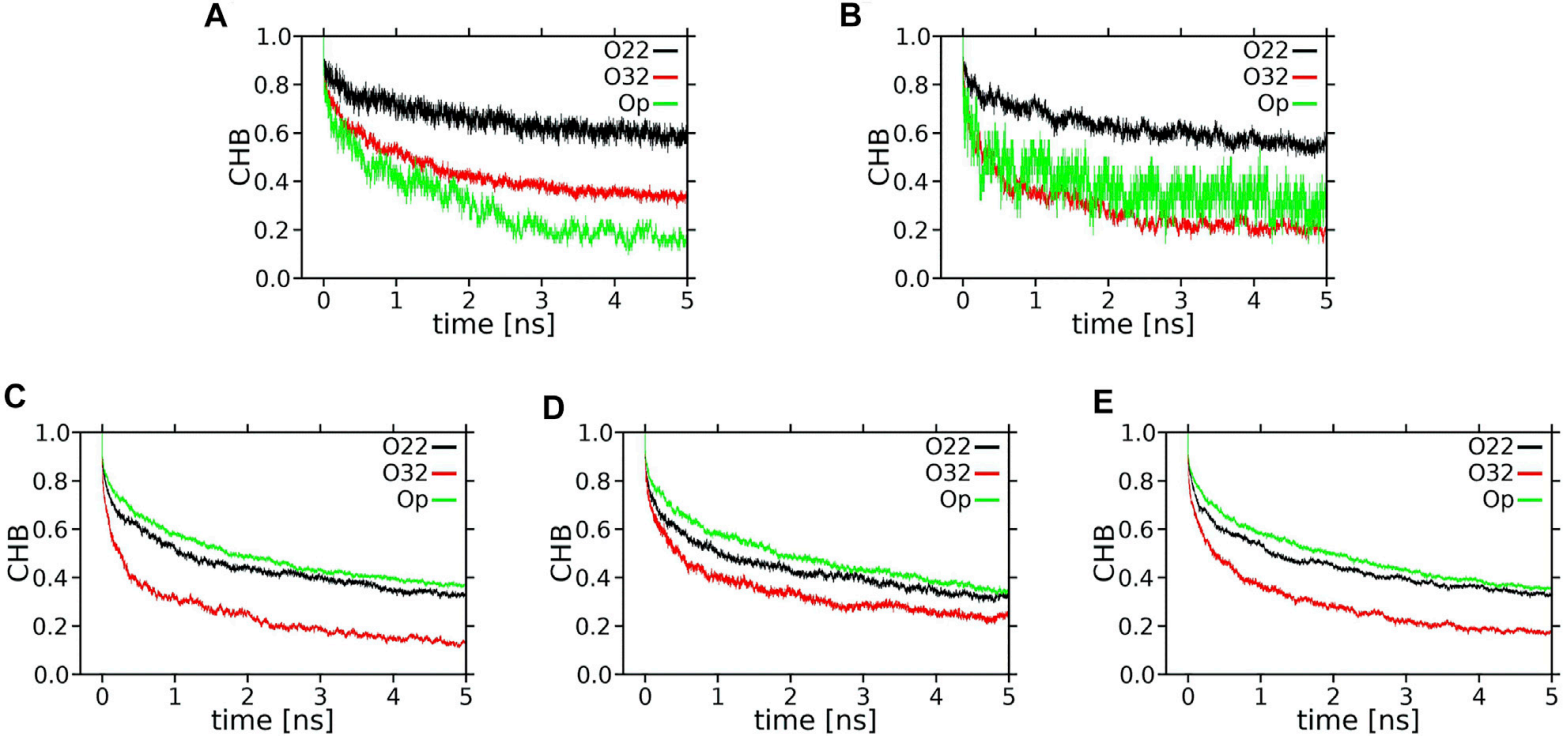
Time profiles (decay curves) of the number of H-bonds between XAN-OH and POPC oxygen atoms (CHB) during the lag time of 5 ns. **(A)** *ε*-ring, **(B)** *ß*-ring, of the horizontal lutein, **(C)** *ε*-ring, **(D)** *ß*-ring, of a vertical lutein, **(E)** *ß*-ring of zeaxanthin (vertical orientation).

Decay curves for XAN-OH···PC H-bonds are shown in Figure 11, while those for XAN-OH—PC water bridges and charge pairs are shown in Supplementary Figure S16. However, because the number of H-bonds is too small (Table 1), which inhibits meaningful averaging and generates a high level of noise in the decay curves, the curves in Figure 11 are not sufficiently smooth for curve fitting. For the curves in Supplementary Figure S16 the situation is similar.

**FIGURE 12:**
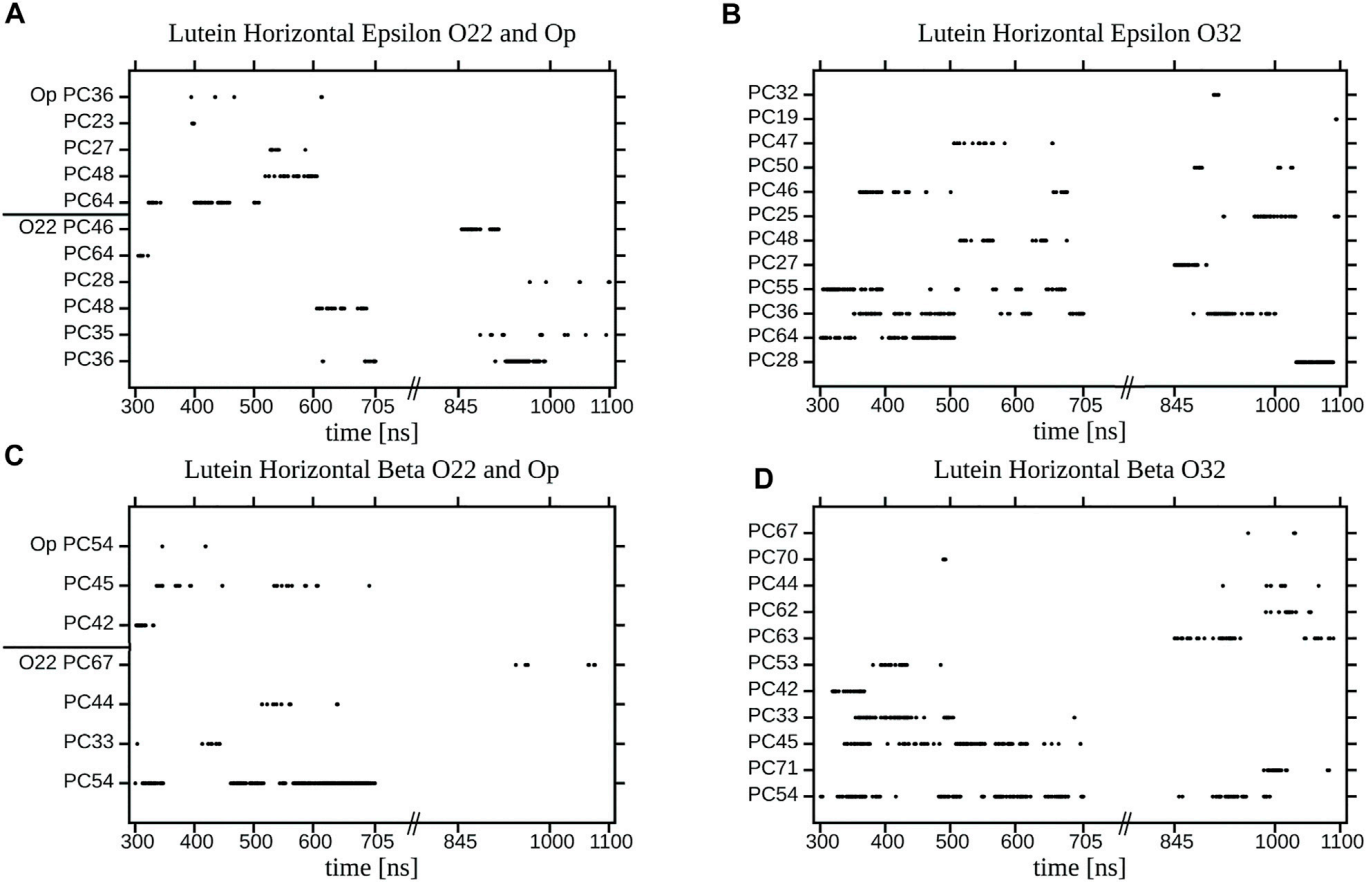
Duration of an individual H-bonding between the OH group of **(A,B)** the *ε*-ring **(C,D)** the *ß*-ring of the horizontal lutein and **(A)** O22 and Op; **(B)** O32; **(C)** O22 and Op; **(D)** O32, atoms of the specified (numbered) POPC molecules in the PC-LUT_H bilayer for two time periods of MD simulation. Points represent firm bonding at a given time.

Each decay curve in Figure 11, because of the way it was calculated (returns are allowed), actually illustrates the decay of the longest lasting H-bonding between two specified atoms. This, however, does not represent an average interaction decay because, as is evident from the panels in Figure 12 and Supplementary Figure S17, there are long periods when the two atoms are not bonded. The panels give accounts of the history of H-bonding between specified POPC oxygen atoms and the *ε* and *ß* rings of lutein in the horizontal (Figure 12) and vertical (Supplementary Figure S17) positions in the bilayer, every ps during ∼800 ns. Time courses of the zeaxanthin *ß*-rings H-bonding are very similar to those of the *ß*-ring of vertical lutein (not shown). In the panels, each dot represents firm bonding at a given time point; the lack of a dot indicates a lack of interaction. The number of dots was used to calculate the average number of H-bonds given in Table 1. In most cases, the firm H-bonding lasts for a short time, although there are periods of time when the bonding lasts for up to 50 ns, e.g., Figure 12A. Even an isolated 50-ns firm H-bonding is not long enough to stabilise the orientation of a XAN molecule in the bilayer during the whole simulation time of 1,100 ns. Instead, a much shorter-lived interaction that re-establishes a multitude of times during the whole trajectory, e.g., Figure 12B, might contribute more to the stability of the XAN orientation, as do XAN-PC water bridges and charge pairs (Supplementary Figure S16; Table 2) together with much more abundant interactions between XAN and the bilayer water (Table 1; Figures 9, 10) as well as XAN-PC nonpolar interactions (Figure 8).

The panels in Supplementary Figure S17 indicate that the process of re-establishing a broken XAN-OH···PC H-bond between a vertical lutein and PC oxygen atoms in the PC-LUT_V bilayer is much more continuous than in the case of the horizontal lutein in the PC-LUT_H bilayer (Figure 12). This certainly contributes to the stability of the vertical orientation XAN in the bilayer.

**FIGURE 13:**
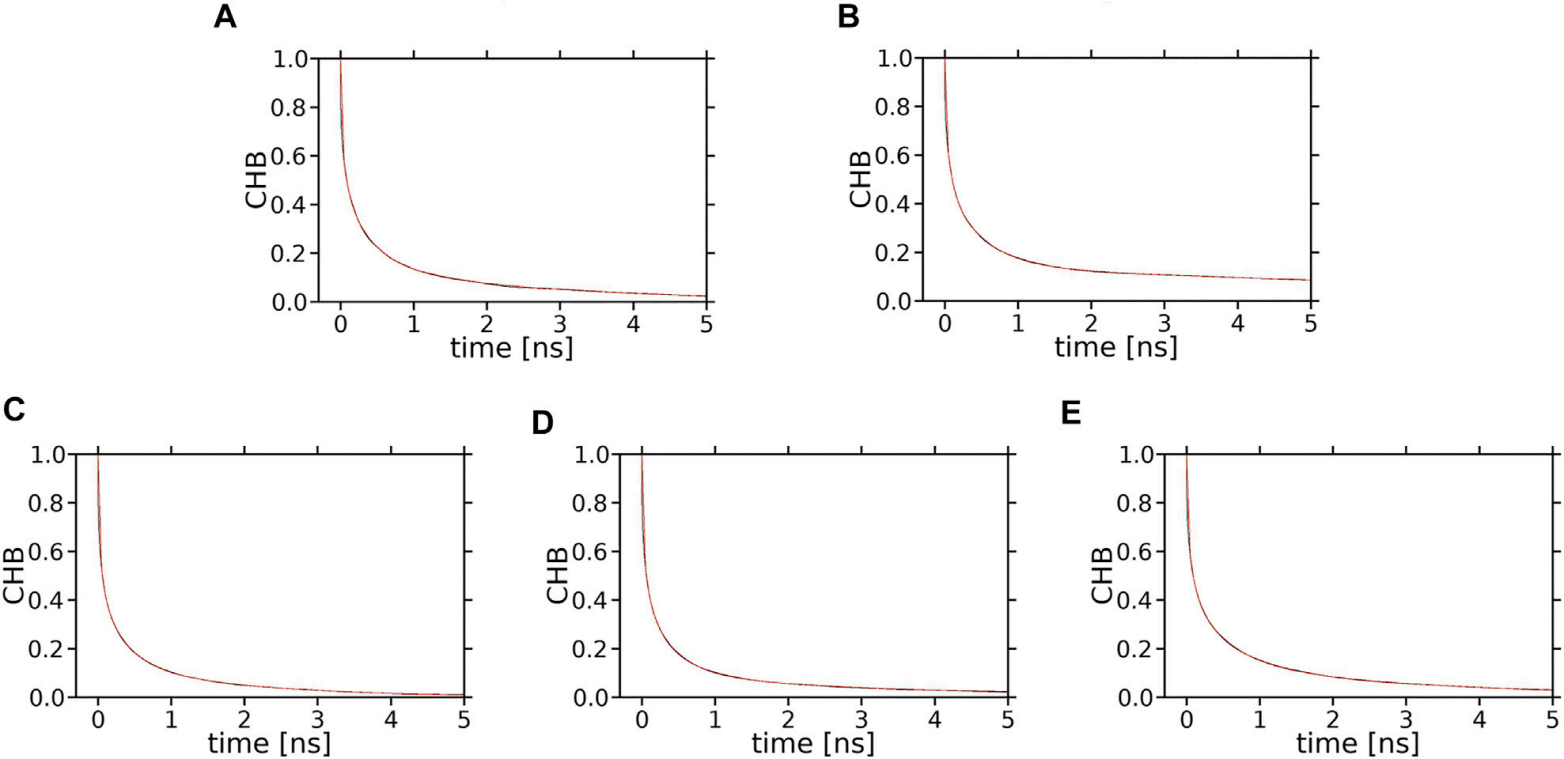
Time profiles (decay curves) of the number of H-bonds between XAN-OH and water molecules hydrating the bilayer (CHB). **(A)** *ε*-ring, **(B)** *ß*-ring, of lutein in the horizontal orientation; **(C)** *ε*-ring, **(D)** *ß*-ring, of lutein in the vertical orientation; **(E)** *ß*-ring, of zeaxanthin in the vertical orientation. Each decay curve (*black*) of the initial number of water molecules H-bonded to the ε-ring or *ß*-ring of lutein or zeaxanthin is fitted to a sum of four exponentials (*red*); the *black* and *red* curves are superimposed and overlap with each other, which indicates that the quality of fits is high.

The number of XAN-PC water bridges and charge pairs (Table 2) is not much larger than that of H-bonds (Table 1), so it is justified to assume that none of the decay curves in Supplementary Figure S16 represent an average interaction decay either. For these reasons the characteristic decay times are assessed numerically neither from the decay curves in Figure 11 nor in Supplementary Figure S16. In contrast, the number of XAN-OH···H_2_O H-bonds in the bilayers indicates that for each XAN-OH group every ps there is ∼1 such a H-bond (Table 1). So, the decay curves for XAN-OH···H_2_O H-bonds shown in Figure 13 represent an average decay process and can be analysed numerically. The decay curves were best approximated with a sum of four exponents. Thus, in the curve fitting procedure the characteristic lifetime, *T*_*i*_, and relative contribution, *A*_*i*_ (pre-exponential factor), of each of the four components in the total decay of H-bonding were obtained. The quality of fits were assessed graphically by calculating residual plots for the curves in each panel in Figure 13. The residue plots shown in Supplementary Figure S18 indicate overall good agreement between each pair of the calculated and fitted curves. The values of *T*_*i*_, and *A*_*i*_ are given in Table 3. The largest contributions of ∼30 and ∼35% to each decay curve have components with intermediate lifetimes of 50–70 ps and 330–500 ps, respectively, (Table 3). Those with very short lifetimes of 1.3–2.2 ps, and very long ones of 1800–9,500 ps, contribute much less, ∼20 and ∼15%, respectively. H-bond lifetimes depend, to some extent, on the type of ionone ring and the XAN orientation in the bilayer. The physical meaning of the components with different time constants and their contribution to the decay of the water···PC H-bonds was deeply discussion in Ref. (Róg et al., 2009). That discussion is also relevant to the water···xanthophyll H-bonds analysed in this paper.

**TABLE 3:** Multiexponential nonlinear fits to decay curves.

	A1	T1	A2	T2	A3	T3	A4	T4
ZEA	22.46 ± 0.06	2.17 ± 0.03	30.52 ± 0.08	72.1 ± 0.4	31.51 ± 0.07	488.7 ± 1.9	15.51 ± 0.05	2981.3 ± 8.9
LUT H *ε*-ring	23.07 ± 0.07	1.35 ± 0.02	27.11 ± 0.10	64.2 ± 0.4	34.41 ± 0.09	396.7 ± 1.6	15.41 ± 0.05	2692.7 ± 7.4
LUT H *ß*-ring	20.06 ± 0.05	1.69 ± 0.02	29.86 ± 0.06	68.0 ± 0.3	35.42 ± 0.05	476.1 ± 1.0	14.65 ± 0.02	9481.5 ± 31.6
LUT V *ε*-ring	23.18 ± 0.06	1.42 ± 0.02	29.96 ± 0.07	50.0 ± 0.2	31.81 ± 0.05	332.5 ± 1.0	15.05 ± 0.04	1808.7 ± 3.3
LUT V *ß*-ring	23.98 ± 0.08	1.84 ± 0.03	31.25 ± 0.09	58.2 ± 0.3	34.44 ± 0.08	380.9 ± 1.3	10.33 ± 0.03	3170.3 ± 11.1

Relative contributions (*Ai*, %) and time constants (*T*_*i*_, ps) of decay curve components for the zeaxanthin *ß*-ring; horizontal lutein *ε*-ring and *ß*-ring; vertical lutein *ε*-ring and *ß*-ring, H-bonds with water in the PC-ZEA_V, PC-LUT_H, PC-LUT_V, bilayers, respectively. The decay curves were fitted to a sum of four exponentials. Errors in the fitted parameters correspond to standard errors.

The intermediate lifetimes of ZEA-OH···H_2_O H-bonds are longer than those of any LUT-OH···H_2_O H-bond. It is interesting to note that the lifetimes of the dominating components of the decay curves in Table 3 are about twice as long as those of PC Oc···H_2_O H-bonds in the PC bilayer derived in Ref. (Róg et al., 2009); this is most likely because the XAN-OH groups are located deep in the bilayer and act as both H-bond donors and acceptors, thus, their H-bonded water molecules exchange with bulk slow.

The number and lifetimes of XAN-OH···H_2_O H-bonds in the bilayers indicate that they contribute mostly to the stabilisation of the orientation of a XAN in the bilayer and that stabilisation in the vertical position of zeaxanthin is greater than that of lutein.

## Results Summary

The results and discussion presented in this paper concern lutein and zeaxanthin whose all torsion angles of the polyene chain are in the *trans* conformation. The dynamics and interactions of these xanthophylls were investigated in the 16:0/18:1 PC (POPC) bilayer and water and also in vacuum. Lutein and zeaxanthin have similar but not identical chemical structures. Both molecules play qualitatively similar functions in living organisms, although small differences in their structures are responsible for their distinct quantitative effectiveness, e.g., (Demmig-Adams et al., 2020a; Widomska et al., 2020). The aim of this study was to correlate differences in the structures of both molecules with their behaviour in the lipid bilayer.

The main structural difference between lutein and zeaxanthin is in their symmetry and the relative orientations of their ionone rings. Both rings (*β*) of zeaxanthin are identical and coplanar with the polyene chain plane. Conversely, the rings (*ε* and *β*) of lutein are different, the *ß*-ring is coplanar with the polyene chain plane, whereas the *ε*-ring is nearly perpendicular to it. As our previous computer modelling study revealed, the orientation of the *ε*-ring perpendicular to the polyene chain plane and the positions of the ring methyl groups, which also interfere with the polyene chain plane, (cf. Figure 9, Ref. (Makuch et al., 2019)) make the intercalation of lutein into the bilayer from the *ε*-ring side less probable than from the *ß*-ring side. The main conclusion of this study is that the orientation of the lutein *ε*-ring is also the supposed reason why a horizontally positioned lutein in the bilayer is less likely to rotate to the vertical position than zeaxanthin. The important factor in this rotation is the fact that the orientation of the polyene chains of both lutein and zeaxanthin in the horizontal position is perpendicular to the bilayer surface (Figures 9, 10). The readiness of a horizontal zeaxanthin to rotate to the vertical position originates most likely from the opposite directions of its *ß*-ring OH groups—one is oriented towards the bilayer core and the other towards the water phase (Figure 9). It should be recalled here that the symmetry of the zeaxanthin molecule refers not only to the identity of its ionone rings but also to its two halves (1–15 and 1–15′, Figure 1) related by C_2_ symmetry. Consequently, the MET groups of each half and the OH group of each *ß*-ring of zeaxanthin point in the opposite directions. This is not the case for the *cis*-zeaxanthin of which both OH groups point in the same direction. This enables the molecule to locate in the bilayer horizontally (Luchowski et al., 2021).

As the polyene chain of lutein is the same as that of zeaxanthin and the molecule has only one *ß*-ring, the orientation of its OH group in a horizontal lutein may point towards either the water phase or the bilayer core with similar probability. At the same time, for both orientations of the *ß*-ring the position of the *ε*-ring in the bilayer is not very different. This, together with an additional obstacle, which is the perpendicularly orientated lutein *ε*-ring, allows to conclude that the rotation of a horizontal lutein to the vertical position is less likely than that of a horizontal zeaxanthin. This conclusion is strongly supported by the result that the probability of the horizontal orientation of lutein in the bilayer is ∼10 times as great as that of zeaxanthin, obtained from the calculated free energy profiles for rotation from the vertical to horizontal position of lutein and zeaxanthin in the bilayer (Grudzinski et al., 2017).

It is interesting to elaborate why one lutein and one zeaxanthin molecule that were apparently trapped in the horizontal positions for ∼500 ns, quite rapidly changed their orientation to vertical. At this point, it should be remembered that the bilayer is a dynamic system and all sorts of motions take place there—their timescales range from very short to quite long (Pasenkiewicz-Gierula and Markiewicz, 2019). As is elucidated in this paper, there are several weak interactions that stabilise the horizontal position of a XAN molecule in the bilayer. Bilayer dynamics may locally disturb the XAN position. Even a small disturbance may lead to breaks in some stabilising interactions, particularly those with water. Imbalance in these interactions may result in molecule rotation. Such rotation can be fast because, in accordance with the free energy profiles calculated in Refs. (Grudzinski et al., 2017; Makuch et al., 2019), the bilayer nonpolar core does not impose much resistance on the cross-bilayer translocation of either lutein *ß* or *ε* ring. Indeed, Figure 7 and Supplementary Figure S8 indicate that crossing of the bilayer nonpolar core by any of the xanthophyll ring is fairly quick which fits well with the result in (Cerezo et al., 2013).

One can wonder if free rotation of a XAN molecule from the horizontal to vertical position ever happens in a real membrane. In this study, in the initial structures of the PC-LUT_H and PC-ZEA_H bilayers, the horizontal position of the XAN molecules was pre-set. However, from our previous molecular modelling study (Makuch et al., 2019) one can infer that such a rotation may indeed happen as one of the lutein molecules that freely intercalated into the PC bilayer positioned itself horizontally and remained in this orientation till the end of MD simulations, which, in fact, was relatively short, lasting 40 ns. The very recent experimental results demonstrated that due to hydrophobic mismatch, *cis*-zeaxanthin may, with hight probability, locate horizontally in the bilayer with its both rings anchored in the interface of the same bilayer leaflet (Luchowski et al., 2021). Light-induced *trans-cis* isomerisation of a xanthophyll torsion angle coupled with the reorientation of the xanthophyll molecule taking place in the human retina is proposed as a crucial regulatory mechanism in protecting the human eye from strong light damage (Luchowski et al., 2021). Our study indicates that vertically oriented xanthophylls do not change their position to horizontal, however, the hydrophobic width of the POPC bilayer of 3.03 ± 0.05 nm is not much greater than the apparent length of the lutein and zeaxanthin measured in the bilayer as the average C3-C3′ distance, of 2.83 ± 0.05 nm. However, the hydrophobic width of a bilayer fluctuates, so locally and temporarily it may become sufficiently larger than the lutein effective length and this might promote cross-membrane rotation particularly of lutein as it has a shorter hydrophobic length than zeaxanthin (Luchowski et al., 2021). An experimental investigation the effect of lutein on the main phase-transition temperature of bilayers consisting of saturated PCs with acyl chains of increasing length showed that for up to 18-carbon atom chains the transition temperature decreased—this indicated that lutein position there was predominantly transmembrane. However, for 22-carbon atom chains the effect of lutein was practically not detected (Wisniewska et al., 2006). This does not exclude the possibility of the horizontal orientation of lutein as our results (Table 1) indicate that the rings of the horizontal lutein are buried deeper in the bilayer nonpolar core than those of the vertical ones and attract less water molecules into the bilayer interface.

A detailed analysis of the conformational behaviour of the *ε*-ring and the *ß*-ring torsion of lutein indicates that the spontaneous isomerisation of the *ε*-ring torsion takes place only in vacuum but not in water and in the bilayer; in water and in the bilayer the *ε*-ring torsion stays in the lower energy conformation of 150°. Thus, the rotation of the *ε*-ring cannot play a part in the horizontal location of lutein in the bilayer because such rotation is hindered. In contrast to the *ε*-ring torsion, the isomerisation of the *ß*-ring torsion takes place in all three environments and the transitions between the low energy conformations of 30° and −30° are very fast.

Nonpolar carotenes can be found in different positions and orientations inside the hydrophobic core of a lipid bilayer, e.g., (Jemiola-Rzeminska et al., 2005). In contrast, positional freedom of all-*trans* XAN molecules in the bilayer is quite restricted. In stabilising the preferential vertical and less probable horizontal orientation of XAN in the bilayer, polar interactions that take place at the bilayer interface play a crucial role. The entries in Tables 1, 2 indicate that interactions between XAN-OH and PC polar groups are not very numerous and generally short-lived (Figure 12 and Supplementary Figure S17). In contrast, XAN-OH···H_2_O H-bonds are much more abundant and that a large portion of them participates in water bridging. Such an attraction of water molecules into the bilayer interface by intercalated lutein was also demonstrated experimentally for PC bilayers consisting of PCs with up to 18-carbon atom saturated acyl chains (Wisniewska and Subczynski, 1998). Important factors in XAN-PC and XAN-H_2_O interactions that stabilise position of the molecule in the bilayer are persistence and continuity. Persistence means here that when a H-bond of the XAN-OH group with a water molecule breaks, the water molecule is instantaneously replaced by another water molecule. Continuity refers here to a process of forming-breaking-reforming of an interaction between the same pair of atoms as illustrated, to some extent, by the case of XAN-OH···O32 H-bonding in Figure 12.

## Data Availability

The original contributions presented in the study are included in the article/Supplementary Materials, further inquiries can be directed to the corresponding author.
