# Supplementary material for: Lutein and Zeaxanthin in the Lipid Bilayer–Similarities and Differences Revealed by Computational Studies
Source: Front Mol Biosci. 2021 Oct 26;8:768449. doi: 10.3389/fmolb.2021.768449 (PMC8575744; doi:10.3389/fmolb.2021.768449)
Supplement: Supplementary file 1 [file DataSheet1.PDF]

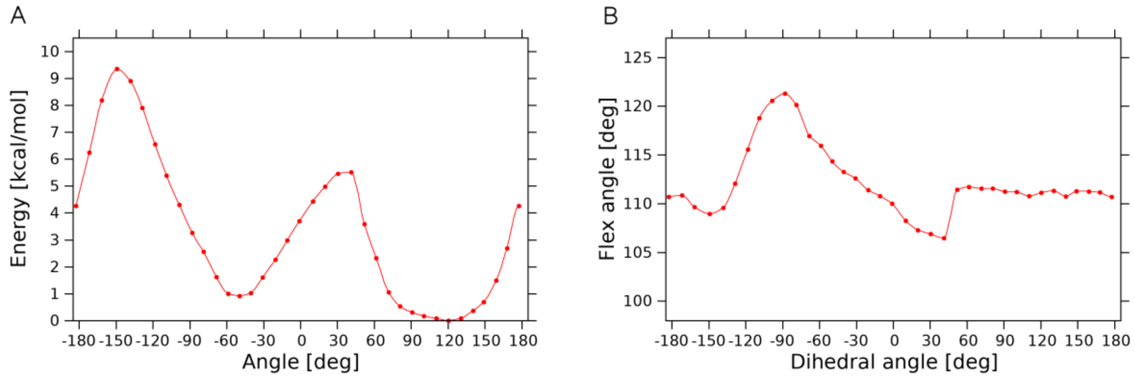

Figure S0. A. the OPLS-AA energy profile for rotation around the C6’-C7’ bond of lutein; B. the flex angle (C3’-C6’-C7’) profile associated with the current value of the C8’-C7’-C6’-C5’ torsion angle. Numbering of the lutein carbon atoms as in Fig 1 of the main text.

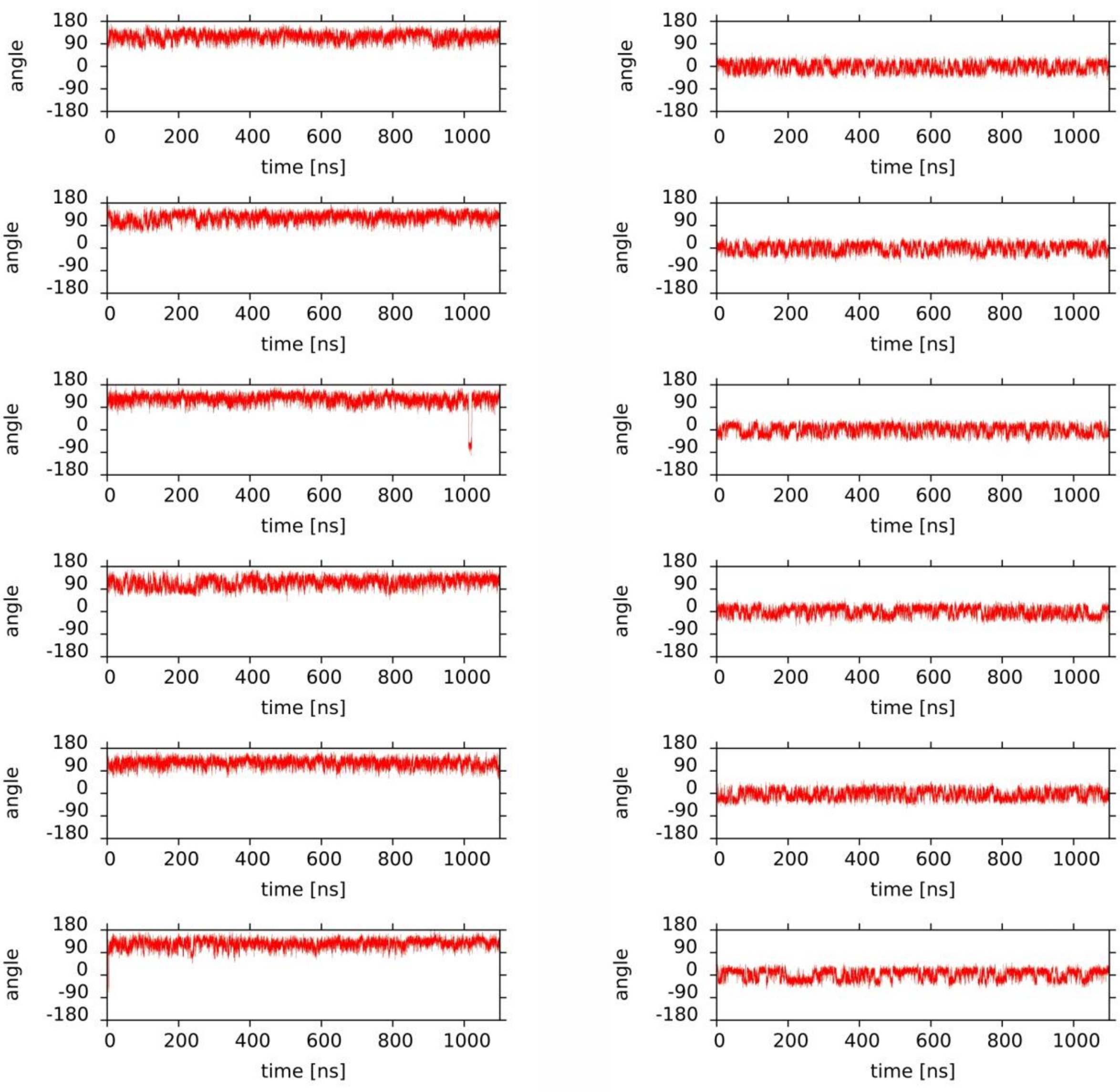

Figure S1. Time profiles of conformations of ε-ring (left) and β-ring (right) torsions of lutein in the PC_LUT_H bilayer.

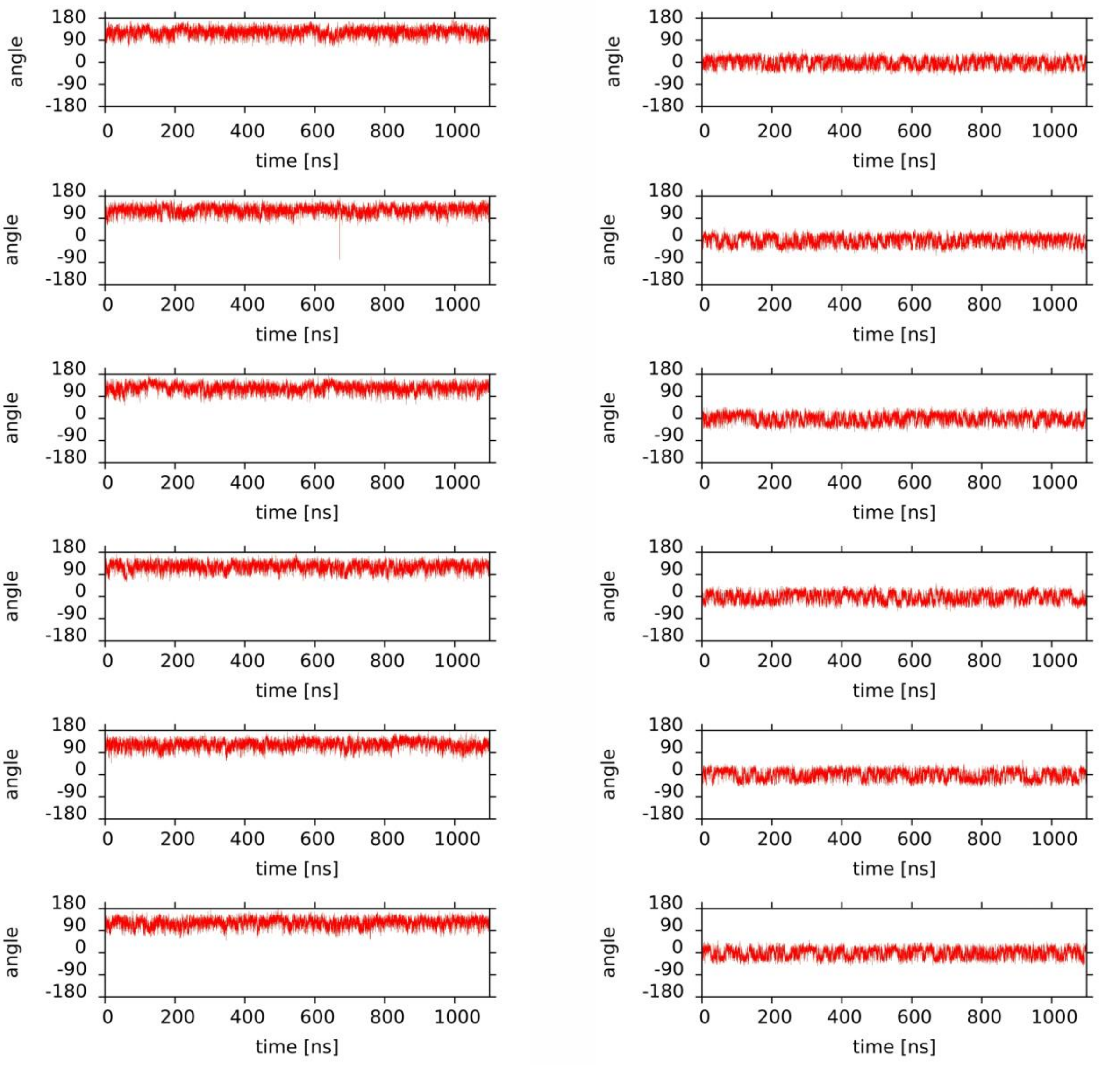

Figure S2. Time profiles of conformations of ε-ring (left) and β-ring (right) torsions of lutein in the PC_LUT_V bilayer.

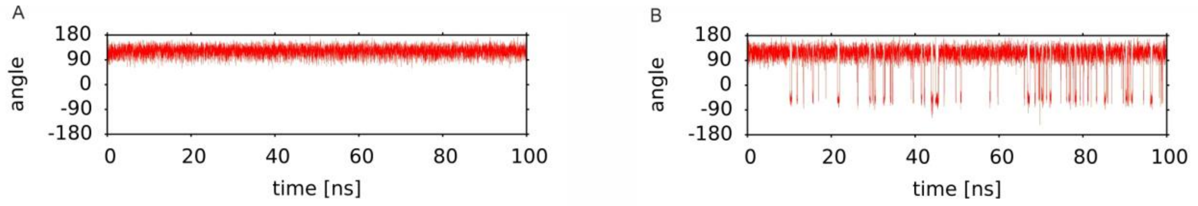

Figure S3. Time profiles of conformations of ε-ring torsions of lutein in (A) water and (B) vacuum.

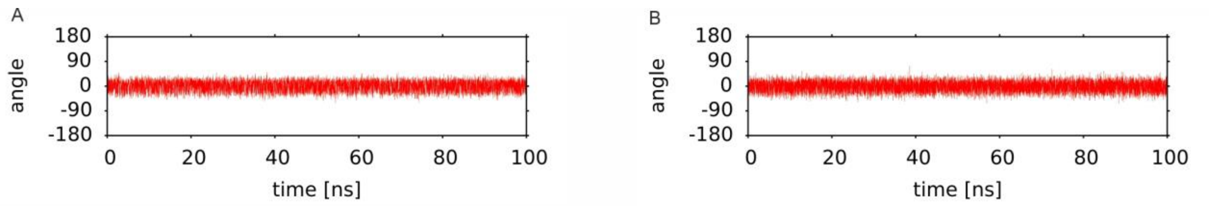

Figure S4. Time profiles of conformations of β-ring torsions of lutein in (A) water and (B) vacuum.

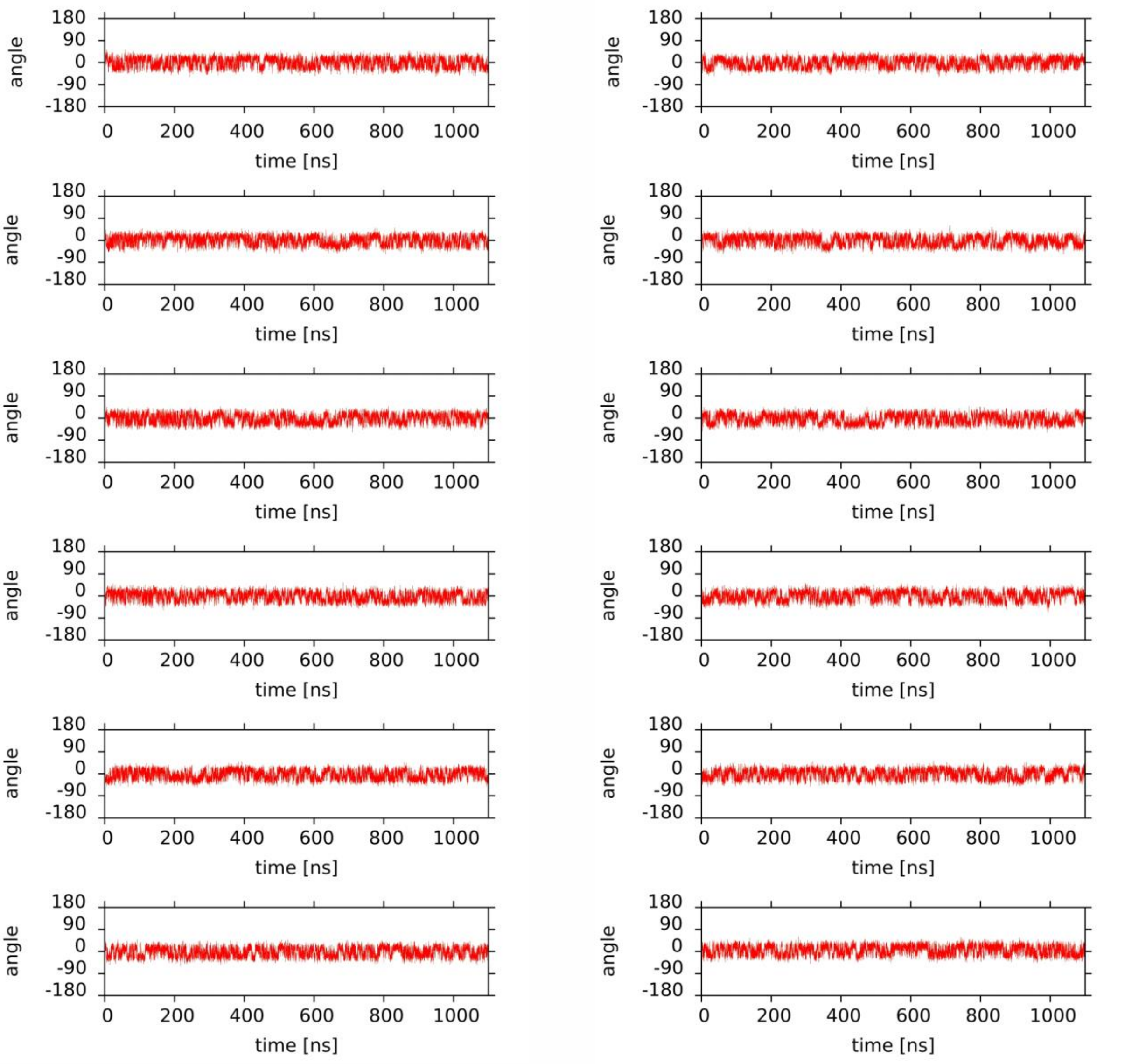

Figure S5. Time profiles of conformations of β-ring torsions of six zeaxanthin in the PC_ZEA_H bilayer (left) and PC_ZEA_V bilayer (right).

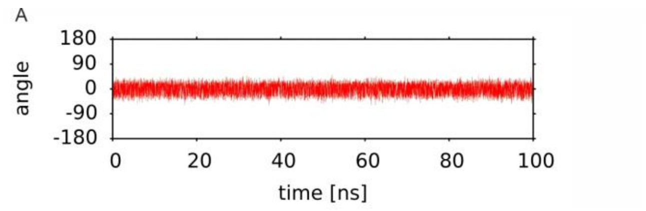

Figure S6. Time profiles of conformations of β-ring torsions of zeaxanthin in (A) water and (B) vacuum.

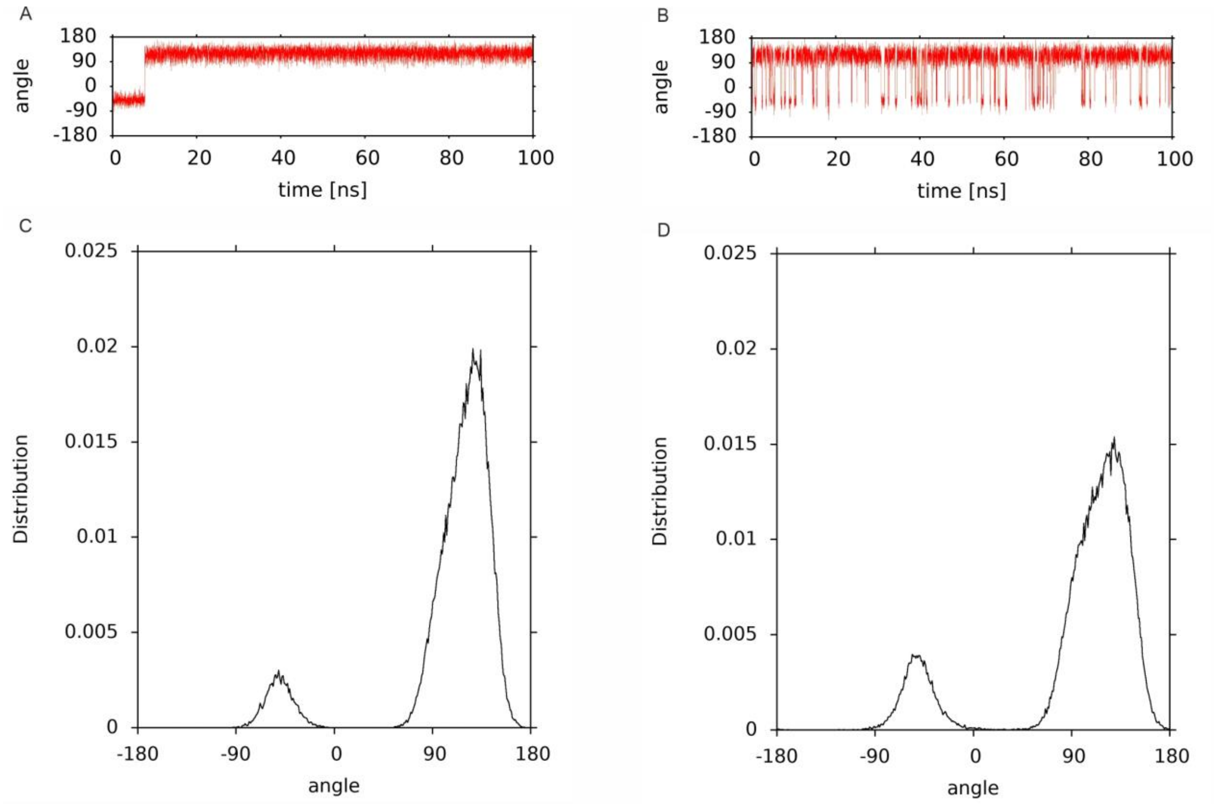

Figure S7. Time profiles of conformations of the ε-ring torsions initially pre-set to –50° of lutein in (A) water and (B) vacuum. (C, D) Population distributions of the ε-ring torsions initially pre-set to –50° in (C) water and (D) vacuum.

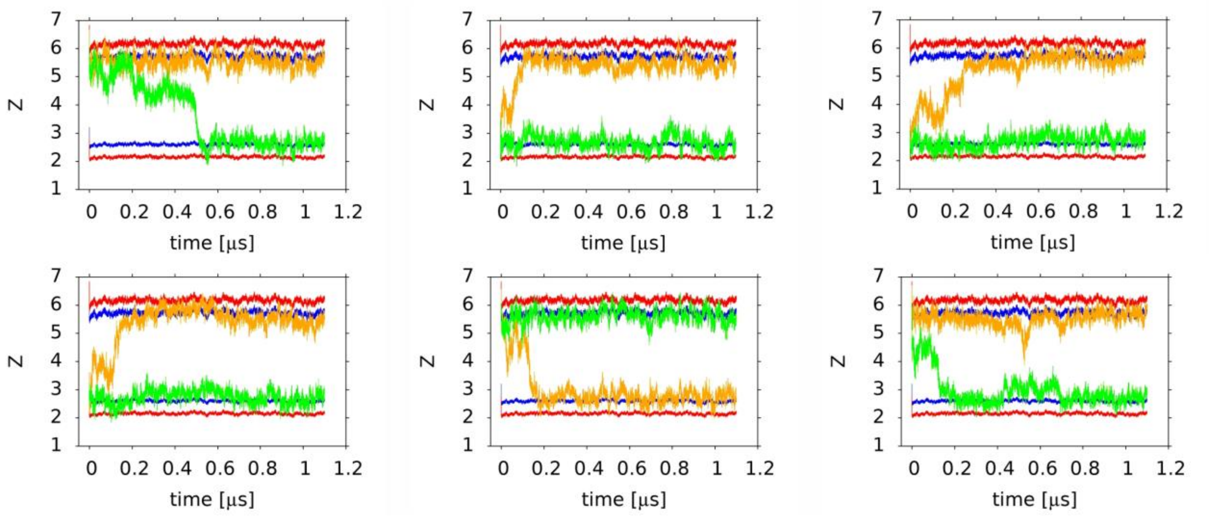

Figure S8. Time profiles of orientations of six zeaxanthin molecules in the PC_ZEA_H bilayer. The *orange* and *green* lines show positions of the centre-of-masses of each of the two β rings relative to the *z*-axis. Two horizontal *red* and *blue* lines indicate the average vertical location of the POPC P and glycerol O atoms in each of the bilayer leaflets.

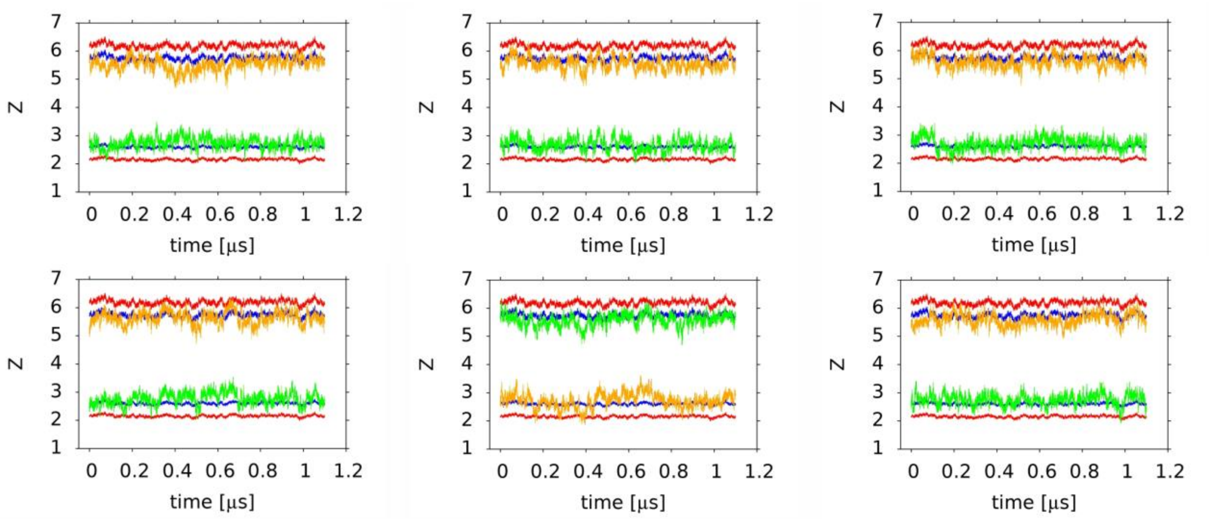

Figure S9. Time profiles of orientations of six lutein molecules in the PC_LUT_V bilayer. The *orange* and *green* lines show positions of the centre-of-masses of the β (*orange*) and ε (*green*) rings relative to the *z*-axis. Two horizontal *red* and *blue* lines indicate the average vertical location of the POPC P and glycerol O atoms in each of the bilayer leaflets.

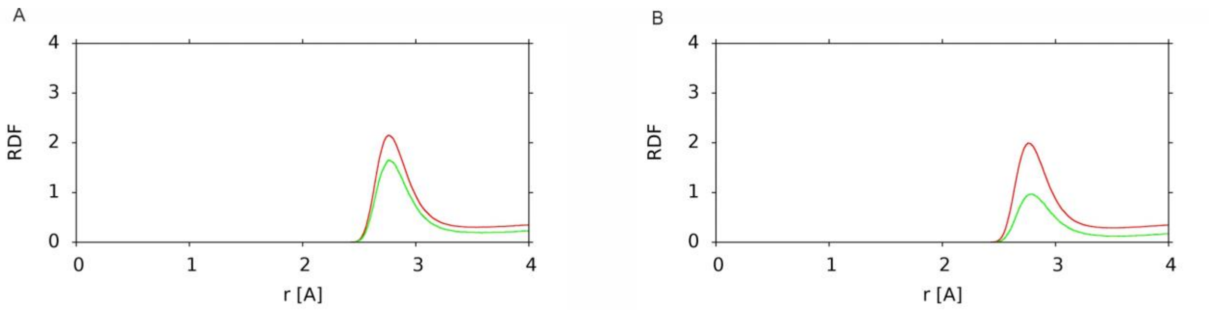

Figure S10. RDF of water molecules relative to the (A) β-ring, (B) ε-ring OH group of a vertical lutein in the PC_LUT_V bilayer (*red*) and the horizontal lutein in the PC_LUT_H bilayer (*green*) . RDFs are cut at 4 Å.

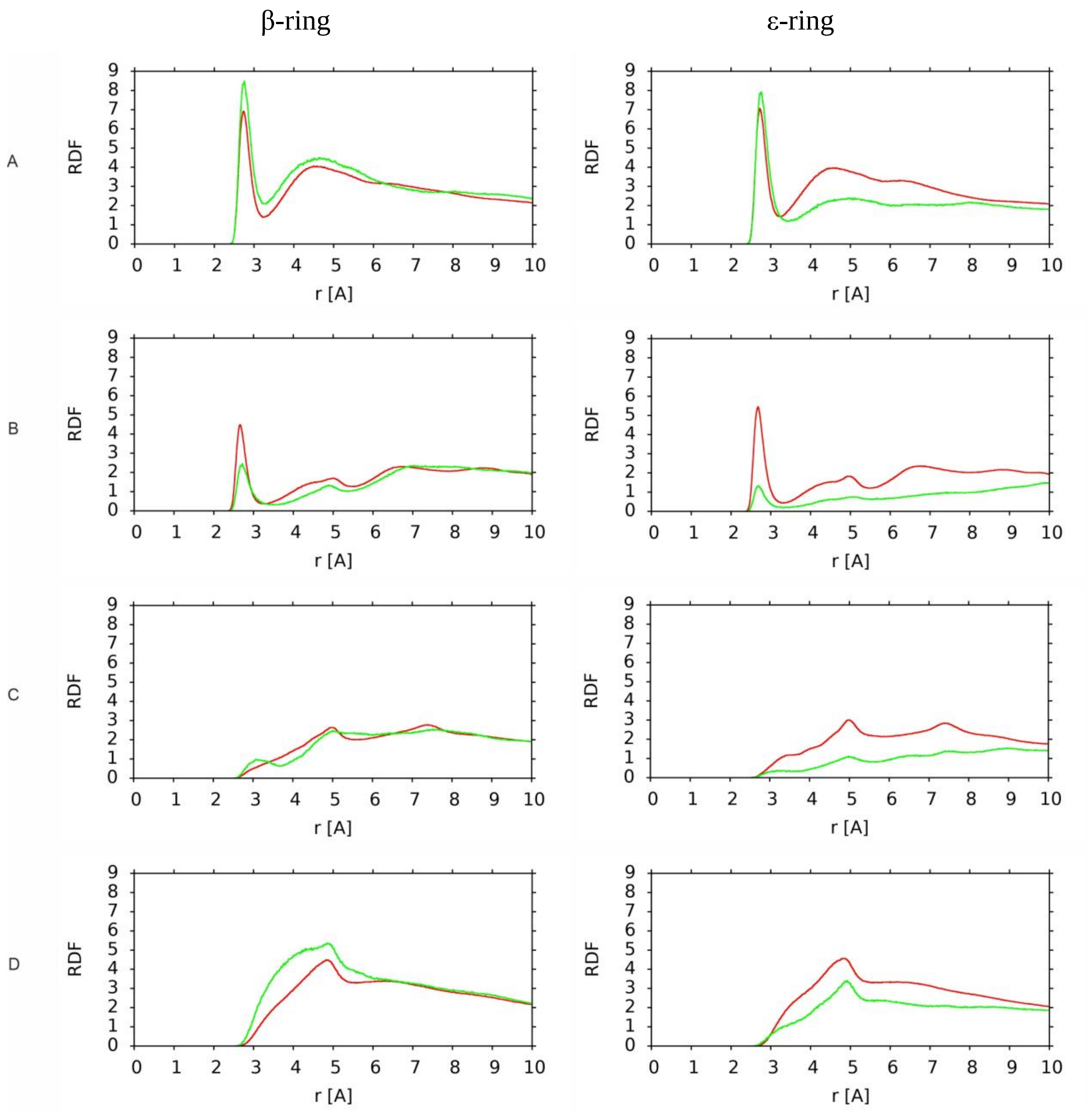

Figure S11. RDF of PC (A) Oc, (B) Op, (C) Oe, (D) Og, oxygen atoms relative to the (left) β-ring and (right) ε-ring of a vertical lutein in the PC_LUT_V bilayer (*red*) and the horizontal lutein in the PC_LUT_H bilayer (*green*). RDFs are cut at 10 Å.

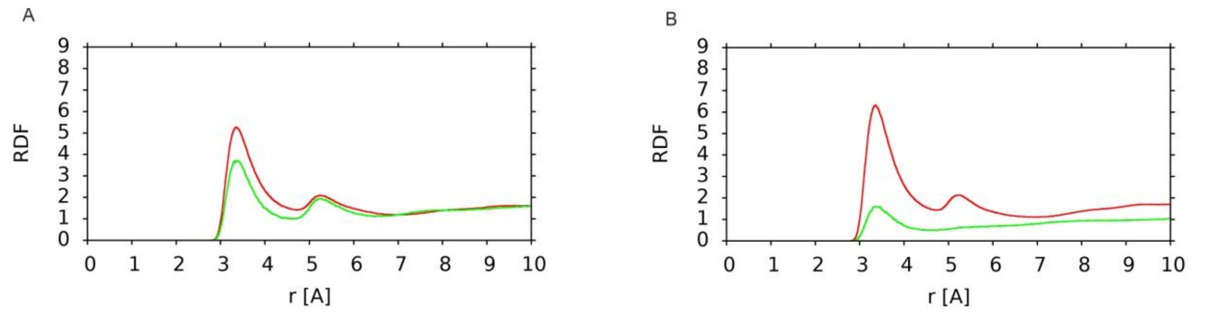

Figure S12. RDF of the lutein (A) β-ring and (B) ε-ring OH group relative to a PC N-CH_3 moiety for a vertical lutein in the PC_LUT_V bilayers (*red*) and the horizontal lutein in the PC_LUT_H bilayers (*green*). RDFs are cut at 10 Å.

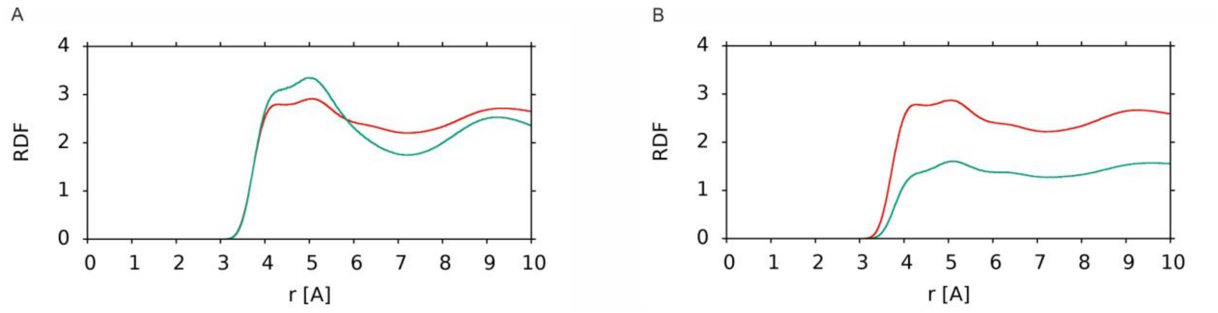

Figure S13. C-C RDF of the XAN-MET (CH_3) groups of the (A) β-ring and (B) ε-ring halves of the lutein polyene chain relative to PC acyl chain carbon atoms for a vertical lutein in the PC_LUT_V bilayer (*red*) and the horizontal lutein in the PC_LUT_H bilayer (*green*). RDFs are cut at 10 Å.

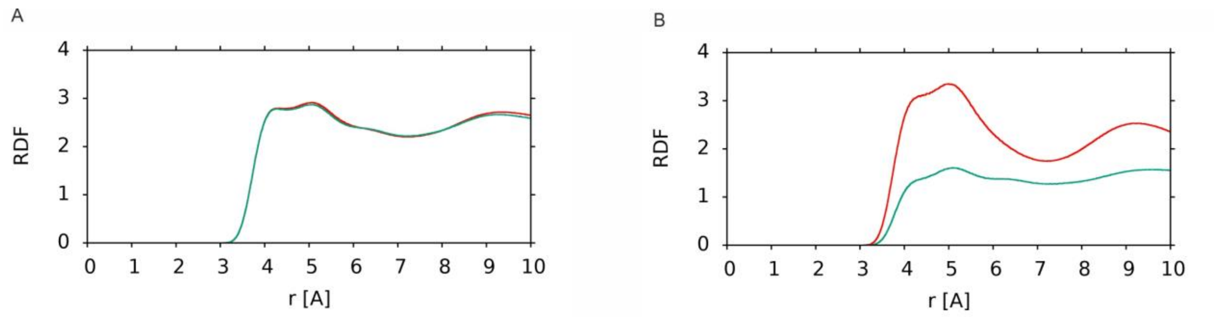

Figure S14. C-C RDF of the XAN-MET (CH_3) groups of the (*red*) β-ring and (*green*) ε-ring halves of the polyene chain relative to PC acyl chain carbon atoms (A) for a vertical lutein in the PC_LUT_V bilayer and (B) of the horizontal lutein in the PC_LUT_H bilayer. RDFs are cut at 10 Å.

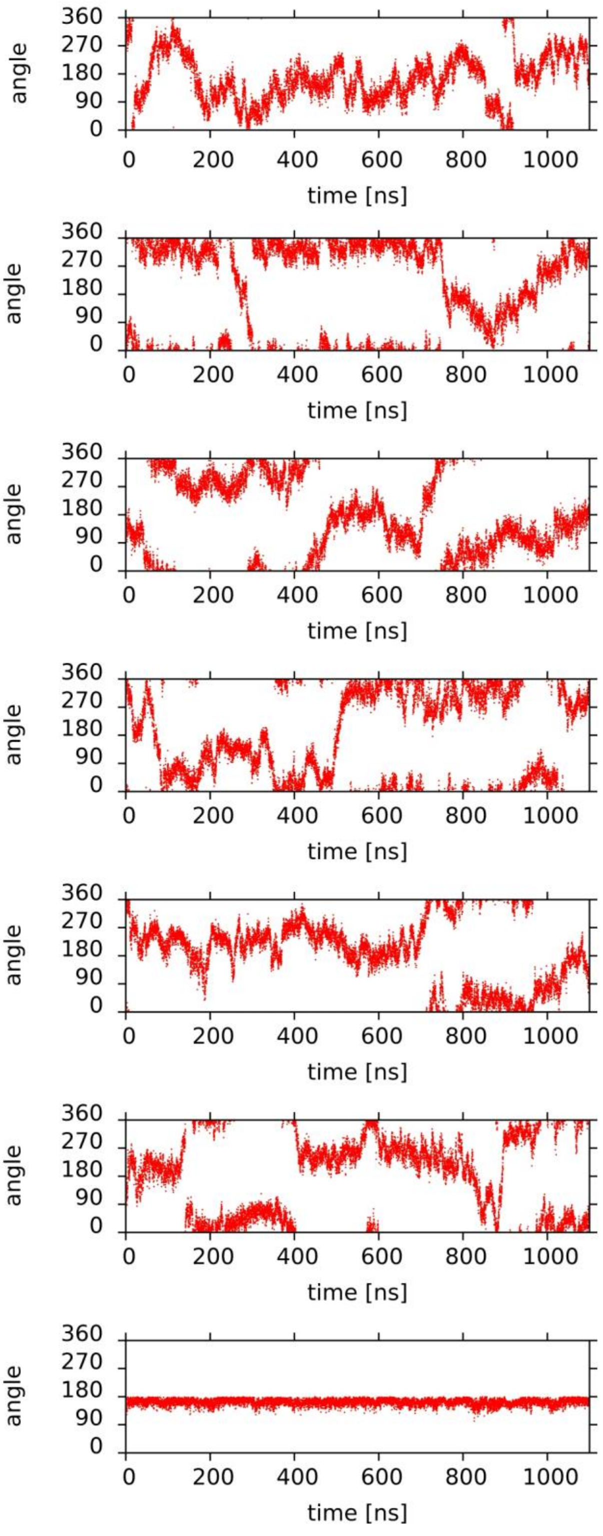

Figure S15. Time profiles of the θ angle of the six lutein molecules in the PC-LUT_V bilayer and (bottom) one lutein molecule (#6) in the PC-LUT_H bilayer that remained in the horizontal position during the whole simulation time. The plots illustrate the rotation of the molecules around their long axes and indicate that molecules in the vertical position make at least one

360° turn during the simulation time, whereas the rotation of the molecule in the horizontal position is hindered.

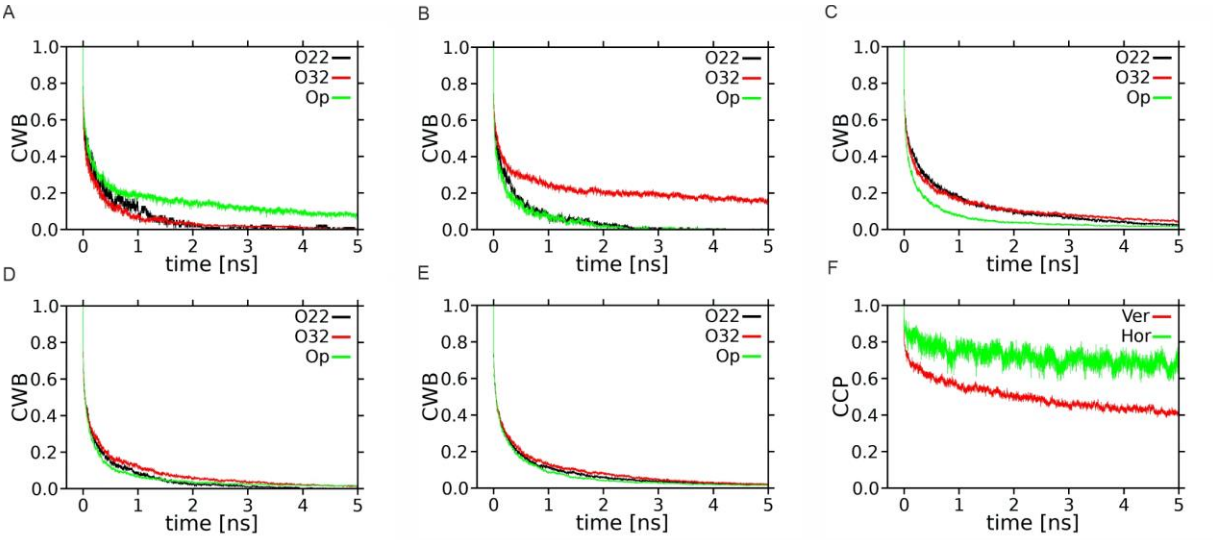

Figure S16. Time profiles (decay curves) of the number of water bridges linking XAN-OH and POPC oxygen atoms, O22 (*black*), O32 (*red*) and Op (*green*) (CWB) during the lag time of 5 ns. (A) ε-ring, (B) β-ring, of the horizontal lutein; (C) ε-ring, (D) β-ring, of a vertical lutein; (E) β-ring of zeaxanthin (vertical orientation); (F) time profiles (decay curves) of the number of charge pairs linking the ε-ring OH and POPC choline groups (CCP) during the lag time of 5 ns, for the horizontal lutein in the PC-LUT_H bilayer (Hor, *green*) and for a vertical lutein in the PC-LUT_V bilayer (Ver, *red*).

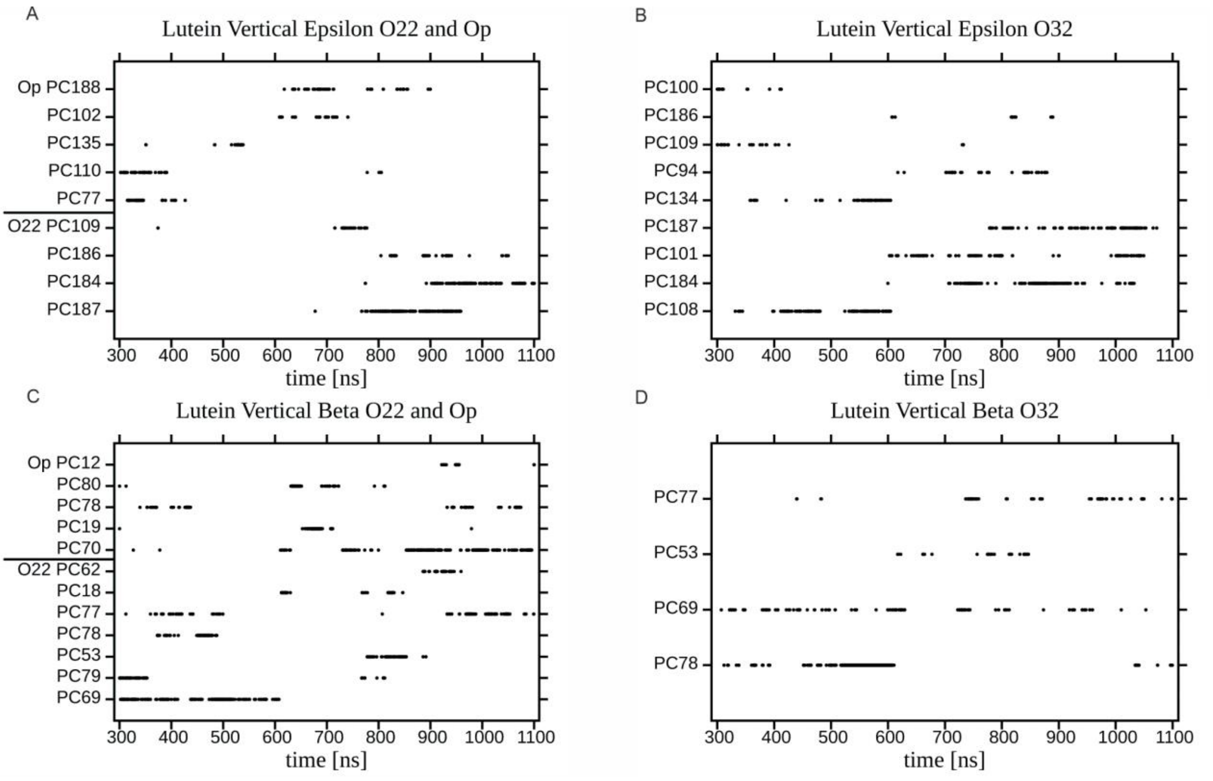

Figure S17. Duration of an individual H-bonding between the OH group of (A, B) the ε-ring; (C, D) β-ring of a vertical lutein and (A) O22 and Op; (B) O32; (C) O22 and Op; (D) O32, atoms of the specified (numbered) POPC molecules in the PC-LUT_V bilayer. Dots represent firm bonding at a given time.

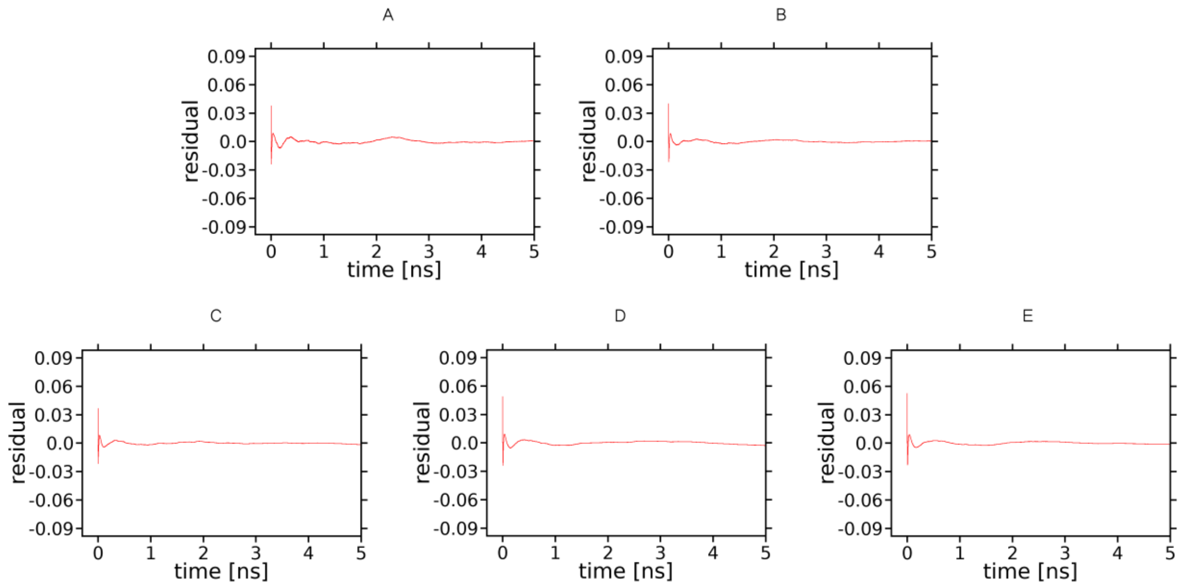

Figure S18. Residual plots obtained by subtracting the calculated from the fitted decay curves presented in the corresponding panels of Fig. 13 of the main text. The curves in Fig. 13 represent time profiles of the number of H-bonds between water molecules hydrating the bilayer and XAN-OH of the (A) ε-ring, (B) β-ring, of lutein in the horizontal orientation; (C) ε-ring, (D) β-ring, of lutein in the vertical orientation; (E) β-ring, of zeaxanthin in the vertical orientation. Except for the initial small (~3%) disagreement between the calculated and fitted decay curves, overall overlapping of both curves in each panel is very good.

Supplementary Film 1 (SF1). The behaviour of six lutein molecules in the POPC bilayer during 1.1-μs MD simulation (only the first 500 ns are shown). In the initial structure, the molecules were placed parallel to the bilayer surface at the depth between the glycerol and the phosphate groups regions. Five of them changed their orientation to vertical in a free rotation but one molecule remained in the horizontal orientation during the whole simulation time. Only the POPC head groups and lutein molecules are shown in the film; the POPC acyl chains, water molecules and the PC head group hydrogen atoms are removed to better show the details of the system. The atoms are represented in standard colours, except for the lutein carbon atoms which are *blue*.

Supplementary Film 2 (SF2). The behaviour of six zeaxanthin molecules in the POPC bilayer during 1.1-μs MD simulation (only the first 500 ns are shown). In the initial structure, the molecules were placed parallel to the bilayer surface at the depth between the glycerol and the phosphate groups regions. All six molecules changed their orientation to vertical in a free rotation. Only the POPC head groups and zeaxanthin molecules are shown in the film; the

POPC acyl chains, water molecules and the PC head group hydrogen atoms are removed to better show the details of the system. The atoms are represented in standard colours, except for the zeaxanthin carbon atoms which are *red*.

The source codes for data analysis and manipulation tools used in this work are available at: https://github.com/krzysztofmakuch/gromacs_tools. They are implemented in Python 3 under the MIT License.
